# SARS-CoV-2 Diagnostics Based on Nucleic Acids Amplification: From Fundamental Concepts to Applications and Beyond

**DOI:** 10.3389/fcimb.2022.799678

**Published:** 2022-03-23

**Authors:** João M. Vindeirinho, Eva Pinho, Nuno F. Azevedo, Carina Almeida

**Affiliations:** ^1^National Institute for Agrarian and Veterinarian Research (INIAV, I.P), Vairão, Portugal; ^2^Laboratory for Process Engineering, Environment, Biotechnology and Energy (LEPABE), Faculty of Engineering, University of Porto, Porto, Portugal; ^3^Associate Laboratory in Chemical Engineering (ALiCE), Faculty of Engineering, University of Porto, Porto, Portugal; ^4^Centre of Biological Engineering (CEB), University of Minho, Braga, Portugal

**Keywords:** SARS–CoV–2, PCR, diagnostics, isothermal amplification, molecular detection, viral sample processing, NAATs, POCTs

## Abstract

COVID-19 pandemic ignited the development of countless molecular methods for the diagnosis of SARS-CoV-2 based either on nucleic acid, or protein analysis, with the first establishing as the most used for routine diagnosis. The methods trusted for day to day analysis of nucleic acids rely on amplification, in order to enable specific SARS-CoV-2 RNA detection. This review aims to compile the state-of-the-art in the field of nucleic acid amplification tests (NAATs) used for SARS-CoV-2 detection, either at the clinic level, or at the Point-Of-Care (POC), thus focusing on isothermal and non-isothermal amplification-based diagnostics, while looking carefully at the concerning virology aspects, steps and instruments a test can involve. Following a theme contextualization in introduction, topics about fundamental knowledge on underlying virology aspects, collection and processing of clinical samples pave the way for a detailed assessment of the amplification and detection technologies. In order to address such themes, nucleic acid amplification methods, the different types of molecular reactions used for DNA detection, as well as the instruments requested for executing such routes of analysis are discussed in the subsequent sections. The benchmark of paradigmatic commercial tests further contributes toward discussion, building on technical aspects addressed in the previous sections and other additional information supplied in that part. The last lines are reserved for looking ahead to the future of NAATs and its importance in tackling this pandemic and other identical upcoming challenges.

## Introduction

SARS-CoV-2 is classified as part of the family *Coronaviridae* and the genus *Betacoronavirus*, which includes two other well-known human pathogens, SARS-CoV and MERS-CoV; moreover it belongs to the subgenus *Sarbecovirus* together with SARS-CoV (Gorbalenya et al., 2020). The disease directly provoked by SARS-CoV-2 would become known as COVID-19 and was rapidly confirmed to be originated from a new strain of severe acute respiratory syndrome-related coronavirus (Gorbalenya et al., 2020). Following more than two years since the beginning of the pandemic, the combat against the virus is mainly supported by widespread testing and mass vaccination (To et al., 2021). SARS-CoV-2 has been subject to mutational events that reinforced the progression of the virus, contributing for increased transmissibility and high morbidity (Becerra-Flores and Cardozo, 2020; Challen et al., 2021). Furthermore, the growing number of vaccinated people can lead to a precocious relaxation in the adoption of preventive measures, like social distancing and frequent sanitation of people and spaces (To et al., 2021). For all these reasons, testing and efficient isolation of suspected and confirmed cases continues to be of paramount importance for tackling the disease.

**Figure 1:**
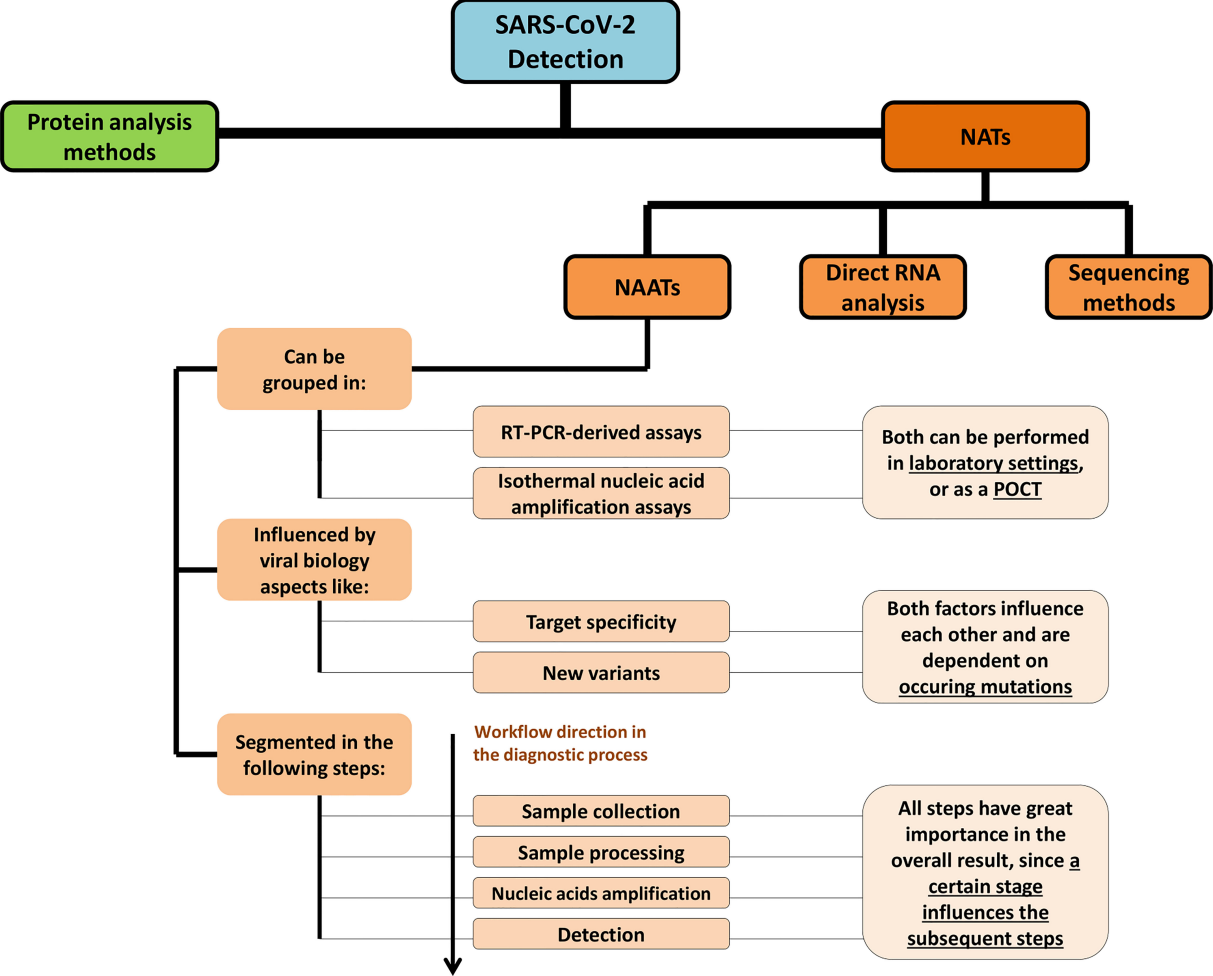
Scheme depicting the different types of NATs, with a particular focus on NAATs. The main characteristics of NAATs are presented, including virology-based factors that influence those tests, the type of amplification routes and amplification settings where these are performed and a brief description of the testing workflow, including its constituting steps.

The diagnostics industry has reached a certain level of maturity that is reflected on the wide range of testing options available, following the continuous development of new solutions since the first days of the health crisis. This effort has been strongly supported by academy and industry, as well as by assessment and certification institutions, which have accelerated the marketing of new *in vitro* diagnostics (IVDs), only turned possible due to exceptional solutions like the dispatch of Emergency Use Authorizations (EUAs), famously provided by Food and Drug Administration (FDA) (Mitchell et al., 2020), in USA. The landscape of SARS-CoV-2 detection comprises molecular analysis tools directed for protein detection like antigen tests (Dinnes et al., 2020) and mass spectrometry-based techniques (Cardozo et al., 2020; Deulofeu et al., 2021), or nucleic acid tests (NATs). NATs can be further divided in nucleic acid amplification tests (NAATs) and other non-NAAT approaches involving the detection of nucleic acids (**Figure 1**). The main application of non-NAAT methods has been focused on sequencing (Harilal et al., 2020; Wang M. et al., 2020; Lu et al., 2020b); moreover, innovative assays for immediate detection of RNA, without an amplification step have also been reported (Moitra et al., 2020; Farzin et al., 2021; Fozouni et al., 2021). While protein antigen tests have an important role in point-of-care testing (Dinnes et al., 2020), the routine diagnosis for the purpose of virus detection is mostly assured by NAATs. The main route for NAAT-based SARS-CoV-2 diagnosis continues to be reverse transcription-real time-polymerase chain reaction (RT-qPCR), which is performed in well-equipped and more and more automated clinical settings (Barra et al., 2020; Nörz et al., 2020). RT-qPCR joins very high sensivity, good specificity and was successfully adapted to the screening of large numbers of samples, what contributed for the implementation of the technique as the gold-standard for SARS-CoV-2 detection and COVID-19 management (Nörz et al., 2020). Isothermal nucleic acid amplification has been intensively explored for point-of-care tests (POCTs) (Bektaş et al., 2021), although being also used in clinical settings, but continues to be less appealing than RT-qPCR (Silva et al., 2021).

This work aims to describe the landscape of NAATs, addressing in the first place associated SARS-CoV-2 virology aspects, such as the rise of concerning mutations and its eventual downstream impact in testing systems; followed by sample collection, including the type of clinical specimens, collection routes, adaptation of high-throughput testing and storage techniques; processing of collected samples, including virus inactivation and RNA extraction procedures; nucleic acid amplification strategies, comprising a detailed description of both PCR-derived and isothermal amplification-based methods, as well as frequently used controls; molecular detection chemistry, which will be separated in those reactions commonly associated with PCR-derived amplification techniques and those linked with isothermal amplification; or platforms for detection, ranging from those traditionally linked with the type of diagnostics used in clinical settings to those integrated in POCTs. The sequence of main topics finally culminates in a benchmark of up-to-date commercial assays. This late stage of the article requests all the knowledge displayed in the former sections for tracing a scenario of the current NAAT-based testing options offered by companies producing IVDs that aim at SARS-CoV-2 detection. The article concludes with a critical commentary on the prospects of NAATs in the near future, focusing on its frailties, necessary improvements and the influence of emerging technologies and new research fields in enhancing the ability of these types of tests to tackle this and coming pandemics.

## Relevant Virology Aspects

### Genome and Virions

**Figure 2:**
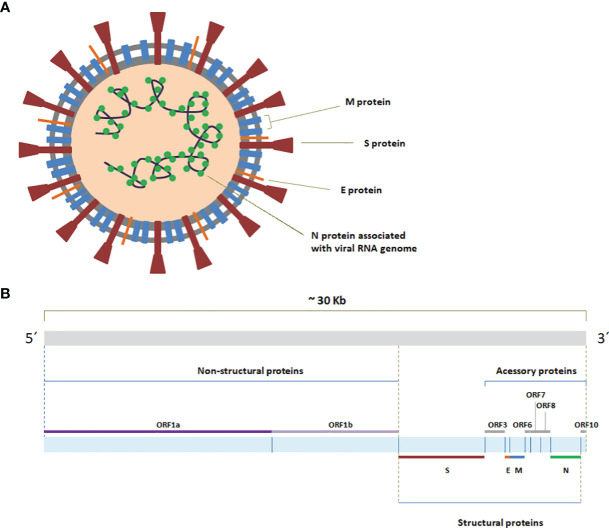
**(A)** Genome organization of SARS-CoV-2. The ORFs constituting the SARS-CoV-2 RNA genome (from 5´ to 3´) encode the non-structural proteins (NSPs), which originate from ORF1a and ORF1b (ORF1ab), the spike (S), envelope (E), membrane(M) and nucleocapsid(N), as well as more than a handful of other dispersed and not-fully characterized accessory proteins (Kim et al., 2020; Hu et al., 2021). The ORFs that lead to accessory proteins include mainly ORF3, ORF6, ORF7, ORF8 and ORF10 (Michel et al., 2020; Giri et al., 2021). **(B)** Schematic representation of SARS-CoV-2 virion.

The genome of SARS-CoV-2 constitutes a long, single and positive sense RNA molecule comprising approximately 30Kb that contains six functional open-reading frames (ORFs) (Kim et al., 2020) (**Figure 2A**). The extremities of the genome are covered with a 5´cap and a 3´poly (A) tail (Miao et al., 2021). ORFs vary considerably in size, with ORF1ab spanning around two thirds of the 5´region of the genome. The path that leads to the production of non-structural proteins (NSPs), from ORF1ab, begins with the entrance of the positive sense RNA molecule in the cell (Kim et al., 2020). There, positive sense RNA undergoes replication that begins with the formation of a negative sense RNA and proceeds with the amplification of positive sense genomic RNA departing from this template (Alexandersen et al., 2020). This newly formed positive sense RNA can be used in the translation of new NSPs, or packed in new virions. ORF1ab is traduced in the two large polypeptides 1a and 1ab respectively, which are then cleaved by viral proteases NSP3 (Kim et al., 2020) and NSP5 (Kim et al., 2020) and originate 16 NSPs in total, with ORF1a originating 11 and ORF1ab leading to 16 (Davidson et al., 2020; Clark et al., 2021). NSP7 and NSP8 in conjunction with NSP12 form the RNA-dependent RNA polymerase (RdRp), a complex of proteins also designated as replicase (Hillen et al., 2020).

The structural proteins of SARS-CoV-2 assemble in virions (**Figure 2B**), in which spike proteins (S) form the characteristic signature in form of halo that is responsible for the prefix “corona” of *Coronaviridae* family (Wertheim et al., 2013). These proteins stem directly from the lipid membrane, exhibiting two subunits and having the adequate configuration for binding with the cell-surface receptor angiotensin-converting enzyme 2 (ACE2), mediating virus entry (Walls et al., 2020; Troyano-Hernáez et al., 2021). Occupying a transmembrane position, the dimeric membrane proteins (M) are the most abundant in the virion and contribute for the maintenance of its shape and support, having a major role in the assembling mechanism, contributing as well for the budding process (Mahtarin et al., 2020; Troyano-Hernáez et al., 2021). Embodied in the membrane, the envelope proteins (E) are the less abundant in the virion and also execute functions related with virion assembling and budding process, in addition to envelope formation (Hassan et al., 2020; Troyano-Hernáez et al., 2021). E protein can form monomeric or pentameric arrangements (De Maio et al., 2020). While forming the pentameric construct it creates an ion channel, classified as a viporin (Cao et al., 2020; De Maio et al., 2020). At last and deeper into the virion, N protein binds to viral RNA genome and organizes it in a helical nucleocapsid structure, the ribonucleoprotein (RNP) complex, interacting also with the M protein in the process of viral assembling and exerting important roles in virus replication and transcription (Troyano-Hernáez et al., 2021; Yang et al., 2021). When it comes to accessory proteins, these molecules execute tasks mainly related to the process of infection, in interaction or within host cells (Mariano et al., 2020; Ren et al., 2020; Flower et al., 2021).

### Viral Variants

The evolution of viruses is driven by the occurrence of changes in the sequence of viral nucleic acids, designated as mutations (Mascola et al., 2021; Nguyen et al., 2021). When a mutation, or group of mutations occurs, it can result in the rise of a variant, following a selection process, over several cycles of replication (Mascola et al., 2021). Generally, those mutations that confer advantage for virus survival lead to its incorporation in the population, through the spread of the new variant in the circulating viruses (Lauring and Hodcroft, 2021). The majority of the SARS-CoV-2 ORFs belonging to circulating strains registered some mutation (Nguyen et al., 2021). ORF1ab (Velazquez-Salinas et al., 2020), and S (Borges et al., 2020; Korber et al., 2020), E, M, N, ORF3a (Issa et al., 2020; Velazquez-Salinas et al., 2020), ORF6, ORF7a, ORF7b, ORF8 (Velazquez-Salinas et al., 2020) and ORF10 sequences have all been linked with mutation occurrences (Nguyen et al., 2021). As of February 2022 there is a myriad of important mutations and derived variants that demand strict surveillance (WHO, 2021). In order to categorize relevant variants, different institutions use distinct terms, according with the level of potential danger. WHO defines Variants of Interest (VOIs), like Lambda and Mu, or Variants of Concern (VOCs), which demand more attention, like Alpha, Beta, Gamma, Delta and Omicron (WHO, 2021). ECDC adds an additional category to the ones adopted by WHO, namely Variants Under Monitoring (VUMs) (ECDC, 2022), which are designated alternatively as Variants Being Monitored (VBM) by the CDC (Centers for Disease Control and Prevention, 2021).

There is an ongoing debate on the possibility of NAATs being significantly affected by these and other upcoming variants, posing challenges to the already existing diagnostics (Artesi et al., 2020; Jain et al., 2021; Khan and Cheung, 2021; Ramírez et al., 2021; Singh et al., 2021). Early studies on the primers and probes used for RT-qPCR, revealed the existence of mutations that hampered the sensitivity of the reverse primer for RdRp described in Charité protocol and the forward primer targeting N gene in Chinese CDC test (Vogels et al., 2020). B.1.1.7 was already associated with impairment in the use of S gene as target in a commercial multi-target RT-qPCR kit by Thermo Fisher Scientific (Ramírez et al., 2021). Another study highlighted the recurrent identification of mutated genomes in the E gene region, when a RT-qPCR commercial kit produced by Roche and targeting this site was used (Artesi et al., 2020). In order to avoid these situations, conserved regions should be used whenever possible. The efficient tracking of such mutations can facilitate the elaboration of new tests, as well as its redesign and adaptation, by altering the sets of primers and probes (Jain et al., 2021), or even the necessary biosafety practices, in case the new variant becomes more dangerous to manipulate. In addition, efforts must be taken in developing more flexible platforms, recurring to multiple targets (Artesi et al., 2020; Ramírez et al., 2021). Contrarily to an entire negative effect, the failure in detecting a specific target may indicate the presence of a well-characterized variant, thus having an odd, but accessible tool for tracking its spread (ECDC, 2020b; Ramírez et al., 2021). Overall, the negative impact of already circulating variants has been contained with success, in NAATs. However, the fact that SARS-CoV-2 has a medium-to-high mutation rate demands continued surveillance, in particular for tests targeting less conserved regions (Artesi et al., 2020; Wang R. et al., 2020; Jain et al., 2021).

## Sample Collection

### Clinical Specimens and Collection Routes

The range of specimens already used for performing the diagnosis of the virus by NAAT is diverse. It includes samples collected from upper respiratory tract (URT) (Zou et al., 2020), such as nasal (NS) (Calame et al., 2020; Zou et al., 2020), mid-turbinate (M-T) (Barat et al., 2021), nasopharyngeal (NP) (Wyllie et al., 2020), throat (TH) (Zou et al., 2020) and oropharyngeal (OP) (Peng L. et al., 2020) regions, as well as saliva (Wyllie et al., 2020); while others are retrieved from lower respiratory tract (LRT) (Yang et al., 2020), such as sputum (Yang et al., 2020), endotracheal fluid (EDF) (Bergrath et al., 2020), or the liquid resulting from a brochoalveolar lavage (BAL) (Yang et al., 2020). The major international health institutions, like CDC and ECDC recommend the use of URT specimens like NP or OP swabs as a first choice, particularly in asymptomatic, mild or moderate disease cases (CDC, 2020a; ECDC, 2020a). LRT are often recommended in severe cases (ECDC, 2020a), or when negative results are verified in URT samples, despite high suspicion of infection (Yang et al., 2020). Saliva has been a favorite research topic, being the main target of countless works, yielding generally good results (Kapoor et al., 2021; Moreira et al., 2021) as well as wide acceptance for the use in commercial assays (Vogels et al., 2021).

In the months following the declaration of a pandemic, technical and human resource requirements to collect infection-related specimens soon became scarce (Yee et al., 2021). This showed that the traditional sample collection made by medical staff wasn’t ideal in the current pandemic (Tu et al., 2020). Furthermore, there is an increased risk of infection by those performing the collection (Tu et al., 2020; Karthik et al., 2020) and a great part of the samples collected demand well trained personnel, which is unavailable during a pandemic, thus resulting in asymmetric, ill performed sample collections that negatively affect the results obtained (Kinloch et al., 2020). The non-invasive, self-collection of specimens has been accessed (Hall et al., 2020) and perfected (Fernández-González et al., 2021), being increasingly adopted to further avoid the aforementioned downsides of traditional sampling (Wehrhahn et al., 2020). Its benefits suit both POCTs (Hanson et al., 2020) and clinically-based tests (Williams et al., 2020; Yee et al., 2021). Saliva is the easiest specimen to be collected this way and despite becoming viscous and difficult to process, it contains acceptable viral loads (Matic et al., 2021). The NS (Hanson et al., 2020; Valentine-Graves et al., 2020) and TH (Therchilsen et al., 2020) samples have also been explored for the same purpose, but the reports on the overall performance of such samples are controversial, in particular when compared with saliva (Hanson et al., 2020).

The more common way of collecting a sample for analysis is by swabbing it with a sterile instrument (Marty et al., 2020), both in traditional RT-qPCR and in a great part of POCT (Zasada et al., 2020). Aspiration is an alternative to swabs and a common way of collecting some LRT samples like endotracheal secretions (Malczynski et al., 2020). Nevertheless, it is prone to technical problems provoked by sample viscosity (Malczynski et al., 2020). Bronchoscopy techniques are also executed, for instance in the extraction of BAL, but demand highly skilled personnel and pose an increased threat of infection for those doing the collection (Ng et al., 2021). The non-invasive collection must be actively performed by the subject under test, while expelling the substance to be analyzed (sputum, saliva, urine, stool, etc.), sometimes after gargling with a saline solution (Goldfarb et al., 2021), to a sterile recipient. The transport and storage of samples bridge the collection and processing moments. In the exact moment after retrieving the sample, it is placed in an appropriate medium designated as viral transport medium (VTM) (Garnett et al., 2020; Rodino et al., 2020). The formulation of this medium can change according with the commercial supplier, but generally comprises a salt-based solution, with a buffer, a carbon source, serum and antibiotics/antifungals. CDC recommends the following recipe: Anderson’s modified Hanks Balanced Salt Solution (8.0 g/L NaCl, 0.4 g/L KCl, 0.05 g/L Na_2_HPO_4_, 0.06 g/L KH_2_PO_4_, 1.0 g/L Glucose, 0.7 g/L NaHCO_3_, 0.2 g/L MgSO_4_.7H2O, 0.14 g/L CaCl_2_.2H_2_O) with 2% v/v heat-inactivated fetal bovine serum, 100 µg/mL gentamicin and 0.5 µg/mL amphotericin B (McAuley et al., 2021). The high demand for this medium led to shortages during the first year of pandemic that rapidly took the laboratories to look for other media options (Garnett et al., 2020; Radbel et al., 2020) and the FDA to recommend these options (Rogers et al., 2020). The suitable alternatives screened included distinct buffers containing different salts and denaturing agents (Radbel et al., 2020; Rodino et al., 2020). The samples should be refrigerated, being placed at 2°C to 8°C up to 72H, or stored at -70°C, for longer periods (Dzung et al., 2021). The sample can remain stored in VTM during long periods (Dzung et al., 2021), although ideally it should be tested as soon as possible without recurring to storage in the freezer (Dzung et al., 2021). Freezing and thawing of samples can be critical in preserving it and repeated freeze-thawing has significant impact on viral RNA levels (Dzung et al., 2021). Nonetheless SARS-CoV-2 RNA samples have been reported to be able to maintain sufficient integrity for RT-qPCR detection, regardless of the temperature (from -30°C (Rogers et al., 2020) to 35°C (Dzung et al., 2021)), during several weeks (Dzung et al., 2021), for distinct specimens (Rogers et al., 2020).

### High-Throughput Testing

The adoption of high-throughput measures that facilitate the scale-up of RT-qPCR-based diagnostic systems, enabling to expand the number of tests performed, but simultaneously limiting the consumption of reagents has been of paramount interest (Eis-Hübinger et al., 2020; Salimnia et al., 2021). Classical sample pooling consists in mixing different individual samples, generating a pool. The pool is tested and in case of a positive result, the individual samples are retested, in order to find what sample yielded the positive result (Gupta et al., 2020; Yelin et al., 2020). Nonetheless, groundbreaking works have tried to simplify the two-stage process (test and re-test) by developing a method able to detect the individual sample yielding the positive result in a certain pool (Shental et al., 2020; Täufer, 2020). The main downside of pooling is a significant decrease in the analytical sensitivity of RT-qPCR tests (Lüsebrink et al., 2020); the pooling protocols lead to dilution of samples, putting in risk the detection of viral RNA in those containing low viral loads (Lüsebrink et al., 2020). Furthermore, well performed specimen collections are also critical for the success of the strategy, since errors can contribute for further dilution of the sample. Saliva has been a favorite target of pooling approaches (Barat et al., 2021; Pasomsub et al., 2021) due to being easily obtained through self-sampling. NP (Torres et al., 2020) have also been extensively accessed. The approach has even been tested with other non-PCR NAATs, with relative success (Ludwig et al., 2021).

## Sample Processing

### Virus Inactivation

SARS-CoV-2 has been categorized as a hazard group 3 pathogen (Patterson et al., 2020; Welch et al., 2020; Burton et al., 2021), demanding the same biosafety precautions already adopted for handling SARS-CoV (Burton et al., 2021) and MERS-CoV (Burton et al., 2021). The infectious form of the virus is as a general rule manipulated in biosafety level 3 (BSL-3) facilities. An exception to these biosafety norms is the processing of specimens collected for the purpose of diagnosing the virus, either in laboratories, or sites devoted to POC testing (Welch et al., 2020; van Bockel et al., 2020; Burton et al., 2021). Since BSL-3 facilities are scarce, the preparation of samples for testing has sometimes been carried in biosafety level 2 (BSL-2) installations (Welch et al., 2020; Genoud et al., 2021), or even in lower biosafety conditions in the case of POC testing (van Bockel et al., 2020; Welch et al., 2020). The inactivation of coronaviruses can be executed with efficiency by different routes, including physical and chemical approaches (Case et al., 2020; Auerswald et al., 2021) (**Table 1**). Nevertheless, the virucidal agents may negatively impact the biomolecules constituting the virions, hampering the performance of certain diagnostic methods (Loveday et al., 2021). In order to avoid the degradation of viral RNA, preservative solutions must be found.

**Table 1:** Comparison of methods used for complete inactivation of SARS-CoV-2 in clinical samples.

	Type	Stage	Protocol/Reagent used		Reference
Virus inactivation	**Heat treatment**	Before direct analysis, or before RNA extraction	Lower temperature, longer duration	Temperature above 80°C, during at least 1 hour;	(Burton et al., 2021)
			Higher temperature, brief duration	Temperatures above 90°C can inactivate samples in a few minutes;	(Burton et al., 2021)
	**Chemical methods**	Transport	VTM	Primestore MTM, 4M GITC/Tx TM, COPAN eNAT;	(Welch et al., 2020; Richard-Greenblatt et al., 2021)
		Before analysis without full RNA extraction, or in the process of full RNA extraction	Non-anionic detergents	Triton X-100;	(Welch et al., 2020)
			Lysis buffer	ATL, VXL, AVL, Phanter fusion specimen lysis tubes, MagNA Pure External LB, RLT, E&O Lab LB;	(Pastorino et al., 2020b; Welch et al., 2020)
			Extraction reagents	Trizol, Trizol LS;	(Patterson et al., 2020)
		Before direct analysis,	Other	TCEP+ EDTA + Heat.	(Rabe and Cepko, 2020)

The use of heat has been seek due to being an easy (Smyrlaki et al., 2020), minimally harmful (Loveday et al., 2021), environmentally safe (Thompson et al., 2021) and low price (Calvez et al., 2020; Smyrlaki et al., 2020; Thompson et al., 2021) solution for sample inactivation. A vast range of temperatures were tested in distinct works, in the interval between 56°C (Auerswald et al., 2021) and 100°C (Jureka et al., 2020), including several intermediate values (Burton et al., 2021; Loveday et al., 2021; Pryce et al., 2021; Thompson et al., 2021), during periods ranging from 1 (Burton et al., 2021) to 90 (Burton et al., 2021) minutes. Heat treatment consistently proved to be efficient in the neutralization of 100% of the virions, when the temperatures used were equal or above 80°C (Burton et al., 2021; Batéjat et al., 2021); while 80°C of temperature required one hour (Patterson et al., 2020) or more (Burton et al., 2021) for inactivating 100% of the infectious particles, 95°C were able to inactivate the sample in only one minute (Burton et al., 2021). The temperatures between 56°C and 80°C also demonstrated some degree of virucidal activity (Burton et al., 2021), since the reduction in the number of infectious particles was sufficient to meet the requirements for considering a factor as a virucidal agent (Pastorino et al., 2020b) [≥4 Log_10_ TCID_50_, according with the European norm NF EN 14476-A2 (Pastorino et al., 2020b)]. The unit used in the context of RT-qPCR for expressing the load of viral RNA is defined as quantification cycle (Cq). Nonetheless, these Cq value is strongly affected by the inactivation conditions of time and temperatures applied to the clinical specimens. Incubation above 90°C during 5 (Burton et al., 2021) or 15 (Pastorino et al., 2020b) minutes was less impacting in the increase of Cq(ΔCq>5) than 60 minutes or more at 80°C (ΔCq>9) (Burton et al., 2021), considering the same viral load. The aforementioned observations suggest that short periods at higher temperatures are preferable.

Chemical methods constitute the other major alternative for inactivating viral samples before NAATs (Richard-Greenblatt et al., 2021). Non-ionic detergents, (Welch et al., 2020; Auerswald et al., 2021), in addition to chaotropic guanidine salts like guanidine thiocyanate (Welch et al., 2020; Auerswald et al., 2021) and guanidine hydrochloride (Welch et al., 2020) are examples of reagents commonly used as chemical inactivating agents. The referred chemicals can be used for inactivating samples at different stages of the workflow, either in transport (Welch et al., 2020; Richard-Greenblatt et al., 2021), or during the RNA extraction process (Patterson et al., 2020; Auerswald et al., 2021), since these can be incorporated in the formulation of VTM (Welch et al., 2020; Richard-Greenblatt et al., 2021), or in other reagents (Patterson et al., 2020; Welch et al., 2020) used to treat the samples. There are at least two commercial VTM, which already proved to completely neutralize the virus. Furthermore, the range of VTM that revealed to have ≥4 Log_10_ TCID_50_, in spite of not being completely inactivating is vast (Welch et al., 2020). In what concerns the inactivation of viral particles in the process of RNA extraction, detergents (Welch et al., 2020), lysis buffers (Welch et al., 2020) and other extraction reagents have been used (Patterson et al., 2020; Welch et al., 2020; Auerswald et al., 2021). While the systematic use of chemical methods as an inactivation strategy before NAATs can be hampered by supply chain disruption (Auerswald et al., 2021), an increasing number of works have proved the utility of such approach, either on its own (Patterson et al., 2020; Welch et al., 2020; Auerswald et al., 2021), or conjugated with high temperatures (Arizti-Sanz et al., 2020; Rabe and Cepko, 2020).

### Viral RNA Extraction

The extraction of viral RNA comprised in clinical specimens is performed to obtain sufficient target RNA available for reverse transcription and cDNA amplification, aiming to improve the sensitivity of the diagnosis (Wozniak et al., 2020), besides avoiding inhibitors of amplification that can be present in transport media (Klein et al., 2020; Graham et al., 2021). It is usually initiated by submitting the samples to detergent treatment, which promotes the disintegration and solubilization of the viral lipid envelope, in addition to the use of chaotropic agents, like guanidinium salts, or non-specific proteases like proteinase K, which provoke denaturation of RNases (Klein et al., 2020; Genoud et al., 2021; Graham et al., 2021). Following the disruption of virions, RNA is separated and purified from the reagents and products resulting on the disintegration of viral particles (Klein et al., 2020). The methods typically used to obtain this separation include liquid phase extraction using organic-aqueous emulsions (Graham et al., 2021) and solid-phase purification using columns with glass fiber, silica (Klein et al., 2020; Graham et al., 2021) and even magnetic beads (Bektaş et al., 2021; Graham et al., 2021). These approaches result in the concentration of viral RNA (Graham et al., 2021). The commercial solutions for performing full viral RNA extraction include sophisticated and automated instruments (Dimke et al., 2021; Genoud et al., 2021), or more simple, labor-intensive and ready-to-use extraction kits (Wozniak et al., 2020; Ambrosi et al., 2021; Dimke et al., 2021) based on extractions with organic solvents, as well as solid-phase purification (Klein et al., 2020; Graham et al., 2021) (**Table 2**). The assessment of these instruments and kits already proved in numerous studies that a full extraction process plays a significant role in maximizing the recovery of SARS-CoV-2 RNA, increasing the sensitivity of the diagnostic process (Israeli et al., 2020; Lübke et al., 2020). The automated systems usually lead to more standardized assays and originate faster results than handmade extractions (Ulloa et al., 2020; Lázaro-Perona et al., 2021). In general, all specimens can be analyzed through the mentioned methods, without much differences in processing. Sputum and other highly viscous samples can be subject to pre-extraction treatment with sputasol (dithiothreitol) (Peng J. et al., 2020), proteinase K (Peng J. et al., 2020), or acetyl-L-cysteine (Peng J. et al., 2020).

**Table 2:** Comparison of distinct strategies for extracting viral RNA from clinical specimens.

	Type	Operation	Stage	Method used		Reagents and equipments	Reference
**RNA Extraction**	**Full**	Automated	Lysis	Buffer containing detergents, caotropic agents, or proteinase K		MagNA Pure External Lysis Buffer (for use with MagNA pure system, Roche), easyMAG Lysis Buffer (for use with EMAG^®^ and NUCLEISENS^®^EasyMAG^®^ system,Biomérieux)	(Hindiyeh et al., 2019)
			Purification	Solid phase purification, with columns, or moving beads		easyMAG Magnetic silica (for use with NUCLEISENS^®^EasyMAG^®^ system, Biomérieux), Viral NA Small Volume kit (used with MagNA Pure 96 DNA), QIAGEN EZ1 Kits (used with EZ1 Advanced XL, Qiagen)	(Hindiyeh et al., 2019; Ransom et al., 2020)
		Manual	Lysis	Buffer containing detergents, caotropic agents, or proteinase K		AVL, VXL, ATL,RLT (Qiagen)	(Pastorino et al., 2020a; Welch et al., 2020)
			Purification	Liquid phase extraction, with organic-aqueous emulsions		Trizol, Trizol LS, or TRI Reagent (Thermo fisher), EXTRAzol (Blirt)	(Wozniak et al., 2020; Ambrosi et al., 2021; Dimke et al., 2021)
				Solid phase purification, with columns, or moving beads	Silica beads	QIA amp Viral RNA Mini Kit (Qiagen)	(Klein et al., 2020)
					Glass fiber filter	High Pure Viral RNA Kit (Roche)	(Wozniak et al., 2020)
					(Silica) magnetic beads	MagMAX Viral RNA Isolation Kit (Thermo fisher)	(Klein et al., 2020)
	**Partial**	Manual	Lysis	Non-anionic detergents	Triton X-100	Common, available through a wide range of suppliers	(Smyrlaki et al., 2020)
					Tween 20	Common, available through a wide range of suppliers	(Smyrlaki et al., 2020)
				APG solution		Common, available through a wide range of suppliers	(Chomczynski et al., 2021)
			RNase inactivation	Proteinase K		Common, available through a wide range of suppliers	(Genoud et al., 2021)
			Purification	Isopropanol		Common, available through a wide range of suppliers	(Graham et al., 2021)
			Other	Heat		_	(Genoud et al., 2021)

While RNA extraction is relevant, it is not indispensable and constitutes a time-consuming step. The great urgency to optimize the duration of diagnostic tests has given rise to an increasing number of papers concerned with extraction and purification-free approaches in NAATs for SARS-CoV-2 detection (Fomsgaard and Rosenstierne, 2020; Panpradist et al., 2021) (**Table 2**); a tendency closely accompanied by commercial approaches (Bordi et al., 2020; Eckel et al., 2020). The protocols without full extraction methods can directly proceed to RT and cDNA amplification stages (Bruce et al., 2020; Israeli et al., 2020; Smyrlaki et al., 2020), or according with literature can simply include the submission of the collected specimens to a lysis buffer containing non-anionic detergents (Israeli et al., 2020; Smyrlaki et al., 2020; Panpradist et al., 2021), an alkaline polyethylene glycol (APG) solution (Chomczynski et al., 2021), isopropanol (Graham et al., 2021), proteinase K digestion (Genoud et al., 2021; Graham et al., 2021), or heat treatment (Fomsgaard and Rosenstierne, 2020; Smyrlaki et al., 2020). Detergents that were already assessed for this purpose include Triton-X 100 and Tween-20, added to specimens in percentages of 0.5% (Panpradist et al., 2021), or 5% (v/v) (Smyrlaki et al., 2020) in the first case and 10% (v/v) (Smyrlaki et al., 2020) in the second. The APG solution screened contains 65% of polyethylene (v/v) and a pH value between 12.2-12.8, being added to samples in a proportion of 1:2 (Chomczynski et al., 2021). Proteinase K was added to virus-containing samples in a range of concentrations ranging from 0.1mg/mL to 1mg/mL (Genoud et al., 2021; Graham et al., 2021). Sputasol is also used in approaches non-contemplating a full extraction protocol as a pre-processing (Lübke et al., 2020) (before heat treatment, lysis, etc.), or even pre-amplification (Wee et al., 2020) reagent. As expected, literature demonstrates that non-extracted, directly analyzed specimens can render insufficient RNA loads, in particular for asymptomatic or mildly symptomatic individuals (Eckel et al., 2020; Israeli et al., 2020). The sole use of lysis buffers containing Triton-X 100 (Smyrlaki et al., 2020; Panpradist et al., 2021), or other solutions like APG (Chomczynski et al., 2021) and isopropanol (Graham et al., 2021) without any subsequent purification stage appears to improve direct analysis of samples. In a similar way to lysis buffers, proteinase K (Chu et al., 2020; Genoud et al., 2021; Graham et al., 2021) and heat treatment above 95°C during 5 minutes are good solutions for situations in which supply chain shortages are verified and extraction kits aren´t available (Fomsgaard and Rosenstierne, 2020; Beltrán-Pavez et al., 2021); these two methods proved more efficient if used in parallel (Genoud et al., 2021). The specimens that constitute the main targets of non-extraction strategies are NP (Cameron et al., 2021; Chomczynski et al., 2021) and saliva (Chomczynski et al., 2021; Lalli et al., 2021), but OP (Merindol et al., 2020), NS (Panpradist et al., 2021) and TH (Fomsgaard and Rosenstierne, 2020) samples, as well as BAL (Lübke et al., 2020) were also assessed with favorable outcomes.

## Nucleic Acids Amplification

### RT-PCR and Derivates

PCR is a simple and elegant reaction based on the action of a DNA polymerase enzyme and a pair of primers, driven by thermal cycles that sequentially provoke the separation of double helix strands, annealing of primers and its extension, forming new double strands, thus yielding exponential DNA amplification (Green and Sambrook, 2019). The standardization of this process, perfected since the 80´s assured its establishment as the dominant method for DNA amplification (Green and Sambrook, 2019). PCR, initially an end-point, non-quantitative analytic technique turned a real-time tracking tool, with the addition of a fluorescent marker, producing fluorescence proportionally to the number of DNA molecules generated (Green and Sambrook, 2019). This change created a merging between amplification and detection steps, enabling to visualize the profiles of amplification associated with a certain product of amplification, or amplicon, turning the process quantitative (qPCR), faster and even more resistant to nonspecific amplification (Green and Sambrook, 2019). In the context of SARS-CoV-2, RT-qPCR, which results from the inclusion of a reverse transcriptase (RT) in the reaction, in order to convert viral RNA in cDNA has been the first option for performing the diagnosis, mainly due to its increased sensitivity and specificity (Bustin et al., 2021). These advantageous characteristics of the RT-qPCR-related approaches had already been proved in the context of the diagnosis of other RNA virus (Bustin et al., 2021). It was easily adapted due to the robustness of the underlying amplification technique and its popularity, being already widely known in the medical and life science laboratories (Bustin et al., 2021).

RT-qPCR is a technology ready for quantitative analysis, but its quantitative potential has often been misused, or neglected in the context of SARS-CoV-2 diagnostics (Bustin et al., 2021; Han et al., 2021). While a yes or no response about the existence of infection has been found enough as technical feedback resulting from routine diagnosis (all EUA-approved assays for SARS-CoV-2 detection are described as qualitative (Cheema and Blumberg, 2021)), the interpretation of results relies on the Cq values, which are an ambiguous quantification route (Bustin et al., 2021; Han et al., 2021). The performance of a RT-qPCR for objective quantification purposes demands the construction of a calibration curve, with quantified standards, including Cq values in function of known concentrations, thus enabling the determination of the viral concentration in a certain sample (Bustin et al., 2021; Han et al., 2021). When Cq values are taken in consideration without establishing a relation with concentration values displayed by standards, diagnosis isn´t straightly quantitative, since the values of viral load are not harmonized (Bustin et al., 2021). Corman, from Charité developed the first protocol in mid-January 2020 following the divulgation of the viral genome sequence (Corman et al., 2020), prompting other WHO referral laboratories to do the same (Etievant et al., 2020). CDC (CDC, 2020b), CCDC (Etievant et al., 2020), HKU (Etievant et al., 2020) and Pasteur institute (Etievant et al., 2020) also developed important protocols, which together with Charité protocol orientated the implementation of a great part of the tests conducted in multiple laboratories around the world, as well as many commercial kits (Etievant et al., 2020). While RT-qPCR was implemented worldwide for SARS-CoV-2 diagnosis using roughly the same method, there is some variation in the basic reagents and guiding protocols used in the amplification reactions by different routine laboratories, including the primers, probes, RT, reaction enhancers and controls beside specific reaction features, such as single and multiple targeting (that is called multiplex, when several sites are amplified at the same time) (Bustin et al., 2021), or the use of nested amplification (Wang J. et al., 2020) (**Table 3**). There were also reports of the use of other techniques that somehow are based on PCR, like digital RT-PCR (Deiana et al., 2020) and qSTAR technology (In, Diagnostic and Only, 2021) (**Table 3**).

**Table 3:** Compilation of strategies of non-isothermal amplification explored for SARS-CoV-2 diagnostic.

	Method		Method´variation	Region targeted	Single or multiple targeting	Duration of amplification (min)	Included in assay issued with EUA	Source
**Non-isothermal amplification**	PCR-based	RT- qPCR	Single target RT-qPCR	ORF1ab, RdRp, N, E, S	Single	50	Yes	(Jung et al., 2020; Park et al., 2020)
			Multiplex RT-qPCR	ORF1ab, RdRp, N, E, S	Multiple	40-50	Yes	(Jung et al., 2020; Kudo et al., 2020; Park et al., 2020; Mancini et al., 2021)
			N-RT-qPCR	ORF1ab, RdRp, N, E, S	Both	50-120	Yes	(Wang J. et al., 2020; Yip et al., 2020; La Rosa et al., 2021)
		RT-dPCR	RT-ddPCR	ORF1ab, RdRp, N, E N	Both	70-170	Yes	(Vasudevan et al., 2021)
			RT - Chip-based dPCR		Single	80	Yes	(Poggio et al., 2021)
	Non-PCR	qSTAR Technology		ORF1a	Single	20	Yes	(In, Diagnostic and Only, 2021)

*Retrieved from the instructions for use of approved diagnostic products available in FDA website (https://www.fda.gov/medical-devices/coronavirus-disease-2019-covid-19-emergency-use-authorizations-medical-.devices/in-vitro-diagnostics-euas-molecular-diagnostic-tests-sars-cov-2).

Nested RT-PCR (N-RT-PCR), is one of the offshoots of traditional RT-PCR used for SARS-CoV-2 diagnostic (Wang J. et al., 2020; La Rosa et al., 2021). It differs from RT-PCR in the fact that there are two runs of PCR, using two independent sets of primers (Yip et al., 2020). The two sets of primers are arranged in such a way that the outer set of primers amplifies a first fragment and the inner set of primers amplifies a second amplicon within the product of the first reaction (Yip et al., 2020). According with different works this strategy can be used to prevent false negatives (Wang J. et al., 2020), or can be adapted for targeting the detection of distinct variants of concern, contributing for an improved tracking of the disease (La Rosa et al., 2021). Another alternative, further away from traditional PCR-based methods has been digital RT- PCR (RT-dPCR) (Poggio et al., 2021). It differs from RT- qPCR in the way of quantifying the products of amplification generated following thermal cycling (Quan et al., 2018). In this type of strategy, PCR solution is partitioned in thousands of aliquots prior to thermal cycling (Quan et al., 2018). This leads to the nonexistence of any DNA copy in some of the fractions; then the portion of aliquots where amplification occurred enable the relative quantification of the target sequence recurring to a Poisson statistic (Quan et al., 2018). Droplet digital RT-PCR (RT-ddPCR) is a particular case of RT-dPCR in which the partitioning is achieved with the production of droplets, creating isolated microreactors through the emulsification of the reactional mixture with immiscible oils (Quan et al., 2018). Chip-based digital RT-PCR (RT-Chip-based dPCR) is another variation of the technique (Poggio et al., 2021). The RT-ddPCR has been the main type of RT-dPCR explored in the context of SARS-CoV-2 detection (Deiana et al., 2020; Suo et al., 2020; de Kock et al., 2021). This variation of the standard RT-PCR aims to solve some problems associated with false negative results (Alteri et al., 2020; Suo et al., 2020), since it exhibits enhanced sensivity (de Kock et al., 2021; Vasudevan et al., 2021). Furthermore, RT-ddPCR proved to be sensitive in the direct detection of viral RNA in specimens, without a RNA extraction step (Deiana et al., 2020; Vasudevan et al., 2021) There is still a distant cousin of PCR-based tests, consisting on a distinct amplification reaction, which is designated as quantitative selective temperature amplification reaction (qSTAR) (In, Diagnostic and Only, 2021). It is a significantly faster approach than other non-isothermal amplification methods, constituting a recent innovation (In, Diagnostic and Only, 2021).

### Methods Based on Isothermal Amplification

The isothermal amplification of nucleic acids comprehends an array of strategies that exclusively make use of enzymes for driving the amplification of DNA, or RNA at a constant temperature (Piepenburg et al., 2006). Addition of a RT possibilities detection of RNA, following its conversion to cDNA in methods originally designed for the amplification of DNA (Dunbar and Das, 2019). These methods avoid thermal cycling and lead to obtaining results in less time than PCR, generally without the need for expensive equipment like thermocyclers (Dunbar and Das, 2019). SARS-CoV-2 pandemic accelerated the maturing of a great part of the strategies relying on isothermal amplification of nucleic acids (**Table 4**), with multiple new diagnostics relying on these methods.

**Table 4:** Compilation of strategies of isothermal amplification explored for SARS-CoV-2 diagnostic.

	Method	Variations	Region targeted	Single or multiple targeting	Temperature of reaction (°C)	Duration of amplification (min)	Included in assay issued with EUA	Source
Isothermal amplification	**LAMP**	RT-LAMP	ORF1ab, S, N,M, ORF3a,ORF7a	Single, or Multiple	60-65	40-60	Yes*	(Schermer et al., 2020; Lalli et al., 2021)
		Mismatch-tolerant RT-LAMP	ORF1ab, S, N	Single	63	50	No*	(Lu et al., 2020a)
		Barcoded RT-LAMP	N	Single	65	60	No*	(Schmid-Burgk et al., 2020)
	**TMA**	_	ORF1ab	Multiple	_	_	Yes*	(Pham et al., 2020)
	**NASBA**	_	S and N	Single, or Multiple	41	35-130	No*	(Xing et al., 2020; Wu et al., 2021)
	**RPA**	RT-RPA	ORF1ab, S, N, E	Single, or Multiple	42	15-30	No*	(Qian et al., 2020; Xia and Chen, 2020; El Wahed et al., 2021; Sun et al., 2021)
	**RCA**	_	ORF1ab	Single	23	5-15	Yes*	(Kim et al., 2021)
	**C2CA**	HC2CA	RdRp	Single	37	90	No*	(Tian et al., 2020)
	**HDA**	RT-HDA	_	_	_	_	Yes*	(Quidel, 2020)
	**EXPAR**	RTF-EXPAR	ORF1ab	Single	50	<5-25	No*	(Carter et al., 2021)
	**SDA**	AMC-SDA	N	Single	55	30	No*	(Zhang et al., 2021b)
	**MCDA**	RT-MCDA	ORF1ab, N	Single or Multiple	65	35-60	No*	(Li et al., 2020; Luu et al., 2021)
	**LAMP/RPA**	Penn-RAMP	ORF1ab	Single	38 (RPA) and 63 (LAMP)	40	No*	(Song et al., 2021)

*Retrieved from the instructions for use of approved diagnostic products available in FDA website (https://www.fda.gov/medical-devices/coronavirus-disease-2019-covid-19-emergency-use-authorizations-medical-.devices/in-vitro-diagnostics-euas-molecular-diagnostic-tests-sars-cov-2).

LAMP (Loop-mediated isothermal amplification) is by chance the most accomplished strategy classified as isothermal amplification of nucleic acids. The apparatus for this reaction includes primers and a DNA polymerase enzyme with strand displacement activity besides DNA template (Thompson and Lei, 2020). Primers (4 or 6) are carefully designed through a somewhat complex process that often requires the use of specific software (Jia et al., 2019). The combination of LAMP with an RT within the reaction of amplification, enable the occurrence of reverse transcription simultaneously with the amplification reaction. Overall, when comparing this method with other isothermal amplification forms, the main advantage is the robustness of results. In the context of SARS-CoV-2 it has proved useful for the establishment of strategies aiming fast detection of the virus (Dong et al., 2021), which now at best takes less than 15 minutes (Fowler et al., 2021), without subduing specificity and sensivity (Dong et al., 2021). It is compatible with direct detection of RNA, without an extraction step (Fowler et al., 2021; Lalli et al., 2021). Method variations include the protocols of mismatch-tolerant RT-LAMP (Lu et al., 2020a), avoiding the occurrence of mismatches during primer hybridization; Penn-RAMP (Song et al., 2021), combining other isothermal method (mentioned below) to achieve nested, two-stage amplification, thus curbing false negatives; or barcoded RT-LAMP (Schmid-Burgk et al., 2020), a tool to turn sequencing more accessible.

RPA (Recombinase polymerase amplification) is another technique aiming at nucleic acids amplification departing from DNA. It requests a pair of primers and the activity of four types of enzymes, three of them originally found in bacteriophage T4, like recombinase, recombinase-mediator protein and single-strand DNA binding proteins (SSBs), or a DNA polymerase with strand displacement activity retrieved from bacteria (Piepenburg et al., 2006). When combined with a RT enzyme, this method enables the detection of RNA targets (Xia and Chen, 2020). The fact that it is a fast amplification process, with a simple amplification chemistry that avoids complex design of primers is a positive asset of this technique (Behrmann et al., 2020; Xia and Chen, 2020).Penn-RAMP is an assay that joins LAMP and RPA in the same strategy. In general, RPA has been widely described in literature reporting SARS-CoV-2 diagnostics, despite the inexistence of a commercial assay based on the technique (Behrmann et al., 2020; Qian et al., 2020; Xia and Chen, 2020; El Wahed et al., 2021; Lau et al., 2021). HDA (Helicase-Dependent Amplification) method is centered in the DNA strand displacement activity of helicase enzyme (Barreda-García et al., 2018). When coupled with reverse transcription, the method enables RNA detection (Barreda-García et al., 2018). Despite this method not being extensively covered in SARS-CoV-2-related literature, there is a commercial detection kit that is based on this technique (Quidel, 2020). MCDA (Multiple cross displacement amplification) is a method that amplifies DNA and makes use of ten primers, targeting ten distinct regions and a DNA polymerase with strand displacement activity (Wang et al., 2015). MCDA is related to LAMP and when compared with it, enables faster results, despite a decrease in sensivity (Luu et al., 2021). This method has been explored for diagnostic of SARS-CoV-2 in a consistent way, being mentioned in various works where it is combined with RT enzyme, despite the inexistence of a commercial assay based on the method (Luu et al., 2021; Zhu et al., 2021).

TMA (Transcription-mediated amplification) is a technique of isothermal amplification of nucleic acids especially suited for the detection of viral RNA, since it naturally includes reverse transcription integrated in the mechanism of amplification. The main advantage of this method is its enhanced sensitivity, in some cases detecting even quantities that can´t be traced with RT-qPCR (Gorzalski et al., 2020). It has been considerably explored for SARS-CoV-2 detection, with successful commercial outcomes (Gorzalski et al., 2020; Trémeaux et al., 2020; Beck et al., 2021). NASBA (Nucleic acid sequence-based amplification) is also targeted for RNA amplification and shares great similarity with TMA, since both join the performance of a RT and a RNA polymerase for generating cDNA intermediates, which are again converted in RNA transcripts by the RNA polymerase, thus prompting another amplification cycle (Wernecke and Mullen, 2014; Yan et al., 2014). It has been investigated for SARS-CoV-2 detection, being used as the basis for RNA amplification in two well reported testing strategies (Xing et al., 2020; Wu et al., 2021). RCA (Rolling circle amplification) is based in the biologic mechanism of rolling circle replication for the production of single-strand DNA (ssDNA) or individual RNA strands through the action of DNA or RNA polymerases (Dunbar and Das, 2019). In this kind of reaction, the circular template is targeted by a specific primer in the origin of replication, which is extended by a RNA, or DNA polymerase, leading to the production of ssDNA or RNA strands (Dunbar and Das, 2019). Circle-to-circle (C2CA) amplification is an independent technique derived from RCA (Tian et al., 2020). The enhanced sensivity is a feature of both techniques, despite the extremely long reaction times (Tian et al., 2020; Chaibun et al., 2021). There has been investigation on the potential of both methods for its incorporation in diagnostic strategies for SARS-CoV-2 (Tian et al., 2020; Chaibun et al., 2021; Kim et al., 2021). RCA was already included in a commercial test (Food and Drug Administration, 2020). EXPAR (Exponential amplification reaction) uses two types of enzymes, a DNA polymerase with strand displacement activity and a nicking endonuclease (NEase) (Reid et al., 2018). EXPAR generate around 10^8^ copies of DNA in a few minutes, thus consistently possibilitating to achieve detection in a record time of less than 5 minutes (Carter et al., 2021). This method hasn´t been much explored in literature, in the context of SARS-CoV-2 diagnostic, despite a single exception (Carter et al., 2021). SDA (Strand displacement amplification) is a technique used for both DNA and RNA amplification that relies on the activity of a NEase and request the use of four primers, in addition to a DNA polymerase with strand displacement activity (Dunbar and Das, 2019). While this strategy hasn´t been used for the establishment of any commercial diagnostic in the context of SARS-CoV-2 detection, it is described for detection of this virus in literature, enabling detection without a reverse transcription step (Zhang et al., 2021a).

### Controls Used for NAAT Diagnostics

The adoption of controls and other reference materials for quality assessment in the diagnostic process is of utmost importance to ensure the reliability and standardization of results (Mitchell et al., 2020; Yan et al., 2020). However, the subject hasn´t been covered so often in the context of SARS-CoV-2, as it would be desired, despite a few meritorious exceptions (Mitchell et al., 2020; Page et al., 2020). Therefore, with a significant diversity of NAATs in addition to RT-qPCR and multiple laboratories devoted to such tasks, it is useful to clarify some notions on the existing types of controls and reference materials, as well as the right situation for using each one (Kessler and Raggam, 2012). As a way to further enter the topic, let´s categorize the controls in two main branches: internal controls or external control (**Table 5**), with both being useful when performing all types of NAATs (Yan et al., 2020), regardless of we are talking about isothermal or non-isothermal methods.

**Table 5:** Compilation of the types of controls and corresponding examples used in the context of NAATs targeted for SARS-CoV-2 detection.

	Classification			Stage screened	Common examples	Source
**Controls**	**External**	Positive	Whole process	Entire workflow	Inactivated whole virus, armored SARS-CoV-2 RNA, VLPs	(Wang et al., 2021; Yan et al., 2021)
			Stage	RNA extraction	Inactivated whole virus, armored SARS-CoV-2 RNA, VLPs	(Wang et al., 2021)
				Reverse transcription	SARS-CoV-2 genomic RNA, *In vitro* transcribed mRNA	(SoRelle et al., 2020)
				cDNA amplification	Plasmid DNA, SARS-CoV-2 genomic RNA, *In vitro* transcribed mRNA	(Petrillo et al., 2020; SoRelle et al., 2020)
		Negative	Whole process	Contamination in the entire workflow	Non-infected, cultured human cell lines	(Petrillo et al., 2020)
				Specificity in the entire workflow	Human specimens infected or spiked with human infecting RNA virus (e.g. Influenza A and B, RSV)	(Lee et al., 2020)
			Stage	Contamination during RNA extraction	Nuclease-free water	(Petrillo et al., 2020)
				Contamination associated with reverse transcription	Nuclease-free water	(Petrillo et al., 2020)
				Contamination associated with cDNA amplification	Nuclease-free water	(Petrillo et al., 2020)
	**Internal**	Positive	Endogenous	RNA extraction	Human cells mRNA (e.g. β actin, RNase P), 18S RNA	(Yan et al., 2020)
				Collection of human samples	Human cells mRNA (e.g. β actin, RNase P), 18S RNA	(Yan et al., 2020)
				Reagent/Equipment malfunction	Human cells mRNA (e.g. β actin, RNase P), 18S RNA	(Yan et al., 2020)
			Exogenous	RNA extraction	Non-pathogenic virus (e.g. AoGV, MS2 phage), armored non-SARS-CoV-2 RNA, VLPs	(Calvez et al., 2020; Hasan et al., 2020; Yan et al., 2020)
				Reverse transcription	Non-SARS-CoV-2 RNA (e.g. PVY)	(Calvez et al., 2020)
				cDNA amplification	Plasmid DNA, non-SARS-CoV-2 RNA (e.g. PVY)	(Calvez et al., 2020)
				Inhibition of cDNA amplification	Non-pathogenic virus, armored non-SARS-CoV-2 RNA, VLPs, non-SARS-CoV-2 RNA, Plasmid DNA	(Calvez et al., 2020; Hasan et al., 2020)
				Reagent/Equipment malfunction	Non-pathogenic virus, armored non-SARS-CoV-2 RNA, VLPs, non-SARS-CoV-2 RNA, Plasmid DNA	(Calvez et al., 2020; Hasan et al., 2020)

External controls are designated as “external” due to being run in other well than that of the sample. These controls can give a quality measure of the entire workflow (Kessler and Raggam, 2012) or of independent stages, such as extraction, reverse transcription and amplification (Yan et al., 2020). External positive controls of the whole testing process, also designated as external run controls or batch controls are ideally inactivated viral samples (Wang et al., 2021; Yan et al., 2021) (either cell cultures or human specimens (Corman et al., 2020)), but since these may be difficult to access (SoRelle et al., 2020; Yan et al., 2021) they can be substituted by synthetic controls simulating the viral particles (Chan et al., 2021; Goncharova et al., 2021; Wang et al., 2021); Virus-Like-Particles (VLPs) (Chan et al., 2021; Wang et al., 2021) and armored RNA (Goncharova et al., 2021; Wang et al., 2021; Yan et al., 2021) technologies enable the mimicry of viral protein structures containing packaged SARS-CoV-2 RNA (Goncharova et al., 2021; Yan et al., 2021). External positive controls can act as nucleic acid extraction controls (Wang et al., 2021), enabling to understand if the nucleic acid extraction step was well executed. Positive controls targeted for validation of reverse transcription and amplification are generally SARS-CoV-2 genomic RNA (SoRelle et al., 2020), or *in-vitro* transcribed RNA (Madala et al., 2021); plasmid DNA is also often solely used as a positive control of the stage in which cDNA is amplified (Petrillo et al., 2020). The external positive controls ideally have a known concentration and act as standards (Zhou et al., 2021). The external negative controls of the whole process are typically cultured, non-infected human cell lines (Park et al., 2020; Petrillo et al., 2020). The assay specificity may be assessed by analyzing human specimens infected, or spiked with other RNA viruses that often infect the human respiratory tract, such as Influenza A and B viruses, or respiratory syncytial virus (RSV) (Corman et al., 2020; Lee et al., 2020). However, the most common external negative control aims to validate the reverse transcription and amplification steps, substituting the extracted RNA samples by water in the reactional mixture; these are commonly called no-template controls (Petrillo et al., 2020; Buck et al., 2021) and enable to further discard contamination (or cross-contamination) and confirm the specificity of the test.

Internal controls are used to rule out eventual problems that can occur within a certain assay, being analyzed inside the same well of the sample. In the context of NAATs constructed for SARS-CoV-2 diagnosis, positive internal controls are second targets, not aimed at SARS-CoV-2 detection, which exist in the specimens under test (endogenous internal control (Wang et al., 2021)), or in whole viruses (Calvez et al., 2020), armored RNAs (Goncharova et al., 2021), RNAs (Calvez et al., 2020) and DNAs (Yan et al., 2020) spiked on purpose in the samples (exogenous internal control (Kavlick, 2018)). The range of problems being checked with these controls include ill executed RNA extraction (Goncharova et al., 2021), reverse transcription and (Petrillo et al., 2020) cDNA amplification (Petrillo et al., 2020), improper reagents (Kavlick, 2018), assay nonspecificity (Kavlick, 2018), or inhibition of amplification (Kavlick, 2018; Hasan et al., 2020) and consequent false negative results (Kavlick, 2018).

The set of controls adopted in the RT-qPCR protocol first established by the Center for Disease Control and Prevention (CDC) back in January 2020 reveals that three types of controls should be included in the usual protocol adopted for testing; namely one positive extraction control (often a positive internal control), an external positive control devoted to screen the quality of the RT and amplification steps, as well as a no-template control (CDC, 2020b). The different commercial tests also include similar controls and have strict specifications on its use, which must be followed. Nevertheless, variations exist in the number and type of controls used in those tests. These differences can be a threat to assay comparability. WHO launched a collaborative study to designate an International Standard (IS) for SARS-CoV-2 RNA and in the aftermath of the initiative an inactivated virus standard was established as IS (Bentley et al., 2020), being currently available for purchase through the National Institute for Biological Standards and Control (NIBSC).

## Molecular Detection Routes

### PCR-Associated Routes

TaqMan probes are fluorescence-producing oligonucleotides that specifically target amplicons generated during RT-qPCR (Navarro et al., 2015) (**Table 6**). These probes hybridize with single strand DNA, being labeled with both a fluorophore and a quencher (Navarro et al., 2015). The mechanism that originates the production of fluorescence relies on the hydrolysis of the hybridized Taqman probe by 5´-3´nuclease activity displayed by Taq DNA polymerase, while extending a new complementary strand in the region where the probe first hybridized with single strand DNA (Navarro et al., 2015). These probes have been incorporated in the protocols designed, by Charité (Corman et al., 2020), or CDC (CDC, 2020b) becoming widely adopted for routine diagnostic due to its great sensitivity and specificity (Marinowic et al., 2021). Molecular beacons are another popular type of oligonucleotide probes used in this context that in contrary to TaqMan aren´t hydrolised, but constitute a stem-loop structure with a fluorophore and a quencher in the extremities, which will generate fluorescence when the probe hybridizes, as the quencher no longer is in the proximity of the fluorophore (Banada et al., 2021). DNA-binding dyes, which emit fluorescence when interacting with DNA double strand (Navarro et al., 2015), such as SYBR Green (Marinowic et al., 2021) or EvaGreen (González-González et al., 2020) have been other common options for sensing DNA in PCR-related protocols (**Table 6**). Nevertheless, this interaction between dyes and DNA is nonspecific and enables less sensitive detection than oligonucleotide probes in RT-qPCR (Marinowic et al., 2021). The use of both SYBR Green and EvaGreen is well described for SARS-CoV-2 detection in RT-qPCR (Marinowic et al., 2021) and ddPCR protocols (Falzone et al., 2020). Eletrochemical detection strategies are also noteworthy in the case of PCR-related methods, through the use of intercalating redox reporters (Nunez-Bajo et al., 2020) (**Table 6**). The intercalating redox reporters function by getting intercalated in the double strand DNA, this way yielding an eletroanalytic output that is directly associated with double strand DNA concentration in the sample under analysis. This type of reaction occurs when operating TriSilix, a lab-on-chip technology that enables to perform the RT-PCR reactions commonly used for diagnosing SARS-CoV-2, at miniature scale (Nunez-Bajo et al., 2020).

**Table 6:** Depiction of distinct physicochemical routes used for performance of SARS-CoV-2 detection.

	Physicochemical detection strategy					Real-time or End-point	Included in assay issued with EUA	Source
**Nucleic acids detection**	**PCR-associated methods**	Optical	Fluorescence	Oligonucleotide Probes	Taqman probes	Real-time	Yes*	(Falzone et al., 2020)
					Molecular beacons	Real-time	Yes*	(Banada et al., 2021)
				DNA-binding dyes	SYBR Green, or EvaGreen	Real-time	No*	(González-González et al., 2020; Toptan et al., 2020)
				CRISPR-based	SENA	Both	No*	(Huang et al., 2020)
		Eletrochemical	Voltametry	Intercalating redox reporters	Methylene blue	Real-time	No*	(Nunez-Bajo et al., 2020)
	**Isothermal amplification**	Optical	Fluorescence	Oligonucleotide probes	Molecular beacons	Real-time	Yes*	(Bhadra et al., 2020)
				DNA-binding dyes	SYBR Green, EvaGreen, or SYTO-82	Both	No*	(Ganguli et al., 2020; Garneret et al., 2021; Marinowic et al., 2021)
				CRISPR-based	SHERLOCK	Both	Yes*	(Patchsung et al., 2020)
					CARMEN	Both	No*	(Ackerman et al., 2020)
					DETECTR	Both	Yes*	(Street and Francisco, 2020)
					ENHANCE	Both	No*	(Nguyen et al., 2020)
					CONAN	Both	No*	(Yoshimi et al., 2020)
					FELUDA	Both	No*	(Azhar et al., 2021)
			Chemiluminescence	HPA		Real-time	Yes*	(Inc, 2021)
			Colorimetry	NPs + oligonucleotide probes	AuNPs	End-point	No*	(Alafeef et al., 2021)
				pH indicators		End-point	Yes*	(González-González et al., 2021)
				DNA-binding dyes	SYBR Green, or EvaGreen	End-point	No*	(García-Bernalt Diego et al., 2021; Lau et al., 2021)
				CRISPR-based	SHERLOCK	End-point	No*	(Patchsung et al., 2020)
			Scattering of light	Angle-dependent light scatter analysis		Real-time	No*	(Day et al., 2021)
		Eletrochemical detection	Amperometry	Naopore Target Sequencing		End-Point	No*	(Ptasinska et al., 2021)
			Voltametry	Intercalating redox reporters		Real-time	No*	(Chaibun et al., 2021)

*Retrieved from the instructions for use of approved diagnostic products available in FDA website (https://www.fda.gov/medical-devices/coronavirus-disease-2019-covid-19-emergency-use-authorizations-medical-devices/in-vitro-diagnostics-euas-molecular-diagnostic-tests-sars-cov-2).

CRISPR (Clustered Regularly Interspaced Short Palindromic Repeats) are DNA sequences encountered in the genome of several prokaryotes, resulting from the infection with mobile genetic elements (MGEs) like bacteriophages, plasmids, or transposons, which can be used by these same prokaryotic organisms for recognizing and destroying similar new sequences of DNA, in subsequent infections (Strich et al., 2021). In order to perform the dismantling of the residues left by bacteriophages, CRISPR are transcribed in CRISPR RNAs (crRNAs), which combine with specific endonuclease enzymes that possess great specificity and target complementary sequences (Strich ey al., 2021). These endonuclease enzymes, known as CRISPR-associated proteins (Cas) are powerful gene editing tools and thus have been extensively explored for enhancing the detection of amplicons resulting from amplification reactions (Sun et al., 2021), in the scope of SARS-CoV-2 diagnostics. SENA (Specific Enhancer for detection of PCR-amplified Nucleic Acids) is a confirmatory test to be used in the aftermath of RT-qPCR (**Table 6**), when the Cq sits between 38 and 40, thus raising doubts about the final diagnosis (Huang et al., 2020). It is based in the activity of Cas12a, a fluorescent ssDNA reporter and two crRNAs that target PCR amplification products. SENA increases the sensitivity of RT-qPCR, enabling a more assertive diagnosis (Huang et al., 2020). This type of approach have been explored to overcome the loss of sensitivity associated with the occurrence of mutations in primer-binding sites, by using a version of Cas12a that is able to tolerate single mismatches when it is combined with crRNAs targeting the virus (Huang et al., 2020).

### Isothermal Amplification-Associated Routes

The methods used for detection of products of amplification resulting from isothermal amplification reactions targeting SARS-CoV-2 include mainly optical (Day et al., 2021; Nawattanapaiboon et al., 2021; Reynés et al., 2021) and electrochemical strategies (Ptasinska et al., 2021) (**Table 6**). Optical detection of the virus can rely on fluorescence (Taki et al., 2021; Wu et al., 2021), colorimetry (García-Bernalt Diego et al., 2021; Nawattanapaiboon et al., 2021; Reynés et al., 2021) and even scattering of light (Day et al., 2021). As it happens for PCR reaction, fluorescence-producing reactions are an important route for detecting these products of amplification optically, both in real-time (Ganguli et al., 2020; Alekseenko et al., 2021) and in end-point (Alekseenko et al., 2021; Sherrill-Mix et al., 2021) contexts. The mechanisms that lead to fluorescence production include intercalating dyes (Alekseenko et al., 2021), specific oligonucleotide probes (Jang et al., 2021; Oscorbin et al., 2021), or enzymatic reactions that culminate in the production of fluorescence (Joung et al., 2020; Sun et al., 2021). Intercalating dyes, such as SYBR Green (Alekseenko et al., 2021) or EvaGreen (Alekseenko et al., 2021), are nonspecific but can be used for both real-time monitoring, or end-point measuring of amplification. The potential of nanoparticles in fluorescence-producing reactions has also been described, namely through the employment of magnetic nanoparticles in separating no-specific amplification products prior to fluorescence reading, thus enhancing established protocols (Dahiya et al., 2021).

Chemiluminescence has also been integrated in detection strategies, following isothermal amplification, in particular in TMA (Inc, 2021). Hybridization Protection Assay (HPA) is a method that allows a chemiluminescence –based readout, which relies on a specific oligonucleotide probe attached to an acridinium ester functioning as a reporter molecule by hybridizing with amplicons generated by TMA reactions (Inc, 2021). DNA-binding dyes like SYBR Green and EvaGreen, although being preferably used in approaches relying in fluorescence reading, can also be adapted for colorimetric detection of DNA following isothermal amplification, since both dyes change colors upon interacting with DNA (Bokelmann et al., 2021). Popular colorimetric reactions used for detecting the virus after isothermal amplification resort to conjugation of colored nanoparticles, like AuNPs, with oligonucleotide probes that upon interacting with specific amplicons hybridize and signal the detection of target, through color change (Alafeef et al., 2021). There are Cas-based detection approaches mentioned above that were accessed with colorimetric readouts (Joung et al., 2020; Patchsung et al., 2020), relying similarly in the interaction of oligonucleotide-labeled AuNPs and DNA amplicons. Other colorimetric alternative tested for SARS-CoV-2 detection after isothermal amplification is color change inducted by pH shift, upon detection of amplified DNA, in minimal buffered media (Rabe and Cepko, 2020).

The scattering of light is a less explored method for DNA detection following isothermal amplification, which was also already used in the detection of this virus (Day et al., 2021). This route turns possible in reactions taking place in emulsions and is based on the principle that the newly generated amplicons adsorb to the water-oil interface, resulting in a diminution in the interfacial tension that traduces in a smaller diameter of the emulsion (Day et al., 2021). Then, since the light scatter intensity is directly diameter-dependent, the accumulation of amplicons resulting of amplification can be detected, by monitoring the light scatter intensity (Day et al., 2021). Eletrochemical sensing has been based on amperometry, by using nanopore target sequencing (Ptasinska et al., 2021),or voltametry through the use of intercalating redox reporters (Chaibun et al., 2021), functioning in a similar way to what was described for the PCR-associated case (Day et al., 2021).

Enzymatic reactions engineered for fluorescence production usually rely on the use of distinct Cas endonucleases (Sun et al., 2021). The referred detection mechanisms are often based on the collateral activity of these endonucleases, which is the ability to cleave any ssDNA, or ssRNA in solution, beside the target sequences (Sun et al., 2021). SHERLOCK (Specific High-sensivity Enzymatic Reporter unLOCKing) is a strategy developed prior to SARS-CoV-2 pandemic, which comprises a first step of isothermal amplification, following reverse transcription and a second moment where the amplified DNA is transcribed in ssRNA by RNA polymerase. Then, the ssRNA is targeted by Cas13a endonuclease coupled with a crRNA that recognizes the newly formed ssRNA (Joung et al., 2020; Patchsung et al., 2020). Following the recognition of ssRNA by Cas-crRNA complex, the collateral cleavage activity of Cas13a enables the breaking of a short ssRNA sequence labeled with a fluorophore and a quencher, producing fluorescence in the process (Joung et al., 2020; Patchsung et al., 2020). CARMEN (Combinatorial Arrayed Reactions for Multiplexed Evaluation of Nucleic acids) is a strategy also based in the action of a Cas13 that enables enhanced detection of multiple targets at the same time, thus facilitating reagent savings and testing scalability (Ackerman et al., 2020). DETECTR (DNA Endonuclease Targeted CRISPR Trans Reporter) is another detection approach created prior to pandemic that relies in a first step of isothermal amplification followed by the action of Cas12a (Sun et al., 2021). Similarly, it also comprehends a reaction driven by a Cas endonuclease activated when cRNAs recognize target RNA, thus leading to the production of fluorescence by breaking a ssDNA reporter sequence containing a fluorophore and a quencher (Sun et al., 2021). ENHANCE (ENHanced Analysis of Nucleic acids with crRNA Extensions) involves crRNA modifications, by extending its 3´and 5´ terminations with ssDNA, ssRNA and phosphorothioate ssDNA (Nguyen et al., 2020). These extensions promote self-catalysis and collateral cleavage activity of Cas12a, thus leading to enhanced specificity in target cleavage (Nguyen et al., 2020). CONAN (Cas3-Operated Nucleic Acid detectioN) requires Cas3 and is also based in the collateral activity of this endonuclease (Yoshimi et al., 2020). FELUDA (FnCas9 Editor Linked Uniform Detection Assay) relies on Cas9 for the direct detection of nucleotide sequences, without the need for the cleavage of reporter molecules, as happens in the aforementioned cases (Azhar et al., 2021; Osborn et al., 2021; Xiong et al., 2021). It aims to be an alternative to collateral cleavage activity approaches, while presenting a simpler design and higher resilience to viral mutations, by being able to detect single nucleotide variants (Azhar et al., 2021; Osborn et al., 2021; Xiong et al., 2021).

## Platforms for Performing Nucleic Acids Detection

### Fully Automated Instruments and PCR Equipment

Fully automated systems enable the analysis of large amounts of samples, in a standardized process that integrates nucleic acid extraction, amplification, detection and processing of results (Mayer et al., 2020; Nörz et al., 2020) (**Table 7**). Despite being considerably expensive, these equipments enable high-throughput testing, being tailored for large laboratory settings (Mayer et al., 2020; Nörz et al., 2020). The types of extraction and amplification methods vary, according with the distinct systems. In fully automated machines, extraction approaches match those referred in **Table 2**. RT-PCR constitutes the standard route of amplification (De Luca et al., 2021), although fully automated approaches based on TMA (Trémeaux et al., 2020), or HDA (Quidel, 2020) exist.

**Table 7:** Comparison of platforms used for performing SARS-CoV-2 detection.

	Point-of-care compatibility	Platforms	Main Physicochemical detection strategy	Other steps needed for complete diagnosis	Typical Setting	Included in assay issued with EUA	Source
**Nucleic acids detection**	**Mostly incompatible**	Fully automated equipments	Fluorescence quantification	No	Large, well equiped clinical settings	Yes*	(Nörz et al., 2020)
		PCR equipments	Fluorescence quantification	Eventually, the extraction step	Well equiped to moderate resource settings	Yes*	(Corman et al., 2020)
		Plate readers	Fluorescence quantification	Yes, eventually extraction and amplification step	Moderate resource settings	Yes*	(González-González et al., 2020)
	**Generally compatible**	Portable PCR equipments	Fluorescence quantification	Eventually, the extraction step	Moderate to low resource settings	No*	(Mendoza-Gallegos et al., 2018)
		Portable Fluorescence readers	Fluorescence quantification	Yes, eventually extraction and amplification step	Moderate to low resource settings	No*	(Ireta-Muñoz and Morales-Narváez, 2020)
		Microfluidics	Fluorescence quantification	Eventually, the extraction step	Moderate to low resource settings	Yes*	(Garneret et al., 2021)
		LFA	Colorimetry	Yes, eventually extraction and amplification step	Moderate to low resource settings	No*	(Xiong et al., 2021)
		Single tube	Fluorescence quantification, or colorimetry	Eventually, the extraction step	Moderate to low resource settings	Yes*	(Arizti-Sanz et al., 2020)

*Retrieved from the instructions for use of approved diagnostic products available in FDA website (https://www.fda.gov/medical-devices/coronavirus-disease-2019-covid-19-emergency-use-authorizations-medical-devices/in-vitro-diagnostics-euas-molecular-diagnostic-tests-sars-cov-2).

The standard qPCR equipments are widely described as expensive (Chaibun et al., 2021; Michel et al., 2021; Panpradist et al., 2021) and impractical for use at the point of care (Panpradist et al., 2021) (**Table 7**). Conventional PCR is more affordable but always demands an end-point reading system, normally agarose gel electrophoresis (Silva Júnior et al., 2021), which adds entropy to the diagnostic process, being difficult to implement in a mass testing scenery (**Table 7**). Nonetheless, there has been an effort toward the adaptation of protocols (Panpradist et al., 2021), and construction of more democratic qPCR machines, by decreasing prices (Mendoza-Gallegos et al., 2018), turning the equipments portable (Mendoza-Gallegos et al., 2018) and miniaturizing the systems (Nunez-Bajo et al., 2020; Qin et al., 2020) (**Table 7**). These more accessible apparels can be adapted to operation at the point of care (González-González et al., 2020; Nunez-Bajo et al., 2020). There are examples of the use of qPCR equipments at the point of care with similar success to the standard systems, although the large majority of the RT-qPCR protocols for diagnostic of this virus are still being performed in standard qPCR machines, in clinical settings (Glen et al., 2021) (**Table 7**). Digital PCR is performed in specific apparels that are also associated with high costs, thus being inaccessible for every laboratory, particularly in settings that perform routine diagnosis (Tedim et al., 2021) (**Table 7**). Nonetheless, the high sensitivity of the technique has attracted increasing interest in the context of research (Tedim et al., 2021).

### Plate Readers, Portable Fluorescence Readers, Luminometers and Single Tube Assays

While qPCR equipment proportionate fluorescence reading, there are various techniques used for detection of SARS-CoV-2, which demand the measuring of fluorescence and can´t be performed in qPCR machines. This includes not only protocols adopting conventional fluorescence plate readers (Sun et al., 2021; Tsou et al., 2021), but also strategies suited for measuring fluorescence at the point of care. Portable, miniaturized systems for detection of fluorescence have been developed, which enable nucleic acid detection in this second case (Ganguli et al., 2020; González-González et al., 2020; Ireta-Muñoz and Morales-Narváez, 2020; Samacoits et al., 2021) (**Table 7**). In addition, there are approaches based on UV-Vis spectrophotometry (Health, 2021), which also demand the use of plate-readers, or in the detection of chemiluminescence, which require the use of a luminometer (Inc, 2021) (**Table 7**). The detection of the virus following isothermal nucleic acid amplification can even be fully carried on in a simple tube, with only the support of a heat source able to maintain the temperature for the amplification reaction to occur and a simple visual inspection of the results after a few minutes (**Table 7**). The adaptation of smartphones as fluorescence readers, by combining its ability for data and image analysis with 3D printed components has also been a suggested option (Samacoits et al., 2021). LAMP (Pang et al., 2020) and RPA (Arizti-Sanz et al., 2020) are approaches already used in this type of NAAT. Furthermore, assays that require additional plate readers or portable fluorescence readers, instead of a smartphone may also be considered single tube assays (Behrmann et al., 2020; Lee et al., 2020).

### Lateral Flow Assay

Lateral flow assay (LFA) enables the detection of analytes in a test strip, or dipstick, by submitting the sample to an unidirectional flow in a liquid medium that promotes the interaction of specific targets with capture molecules immobilized in a solid surface (Jauset-Rubio et al., 2016). The platform is designed such that immobilized molecules in a membrane recognize the signature of specific targets, thus yielding a positive or negative colorimetric result (Jauset-Rubio et al., 2016) (**Table 7**). There is one variation of the method, designated as nucleic acid lateral flow assay (NALFA) that is the main route used for end-point, point-of-care detection of DNA amplicons obtained by isothermal amplification, due to a combination of low cost and simple operation requirements (Ivanov et al., 2020). To this point, LAMP (Nguyen et al., 2020), NASBA (Wu et al., 2021), RPA (Azhar et al., 2021; Xiong et al., 2021), or MCDA (Zhu et al., 2021) are isothermal amplification methods already combined with a detection step that comprises LFA, for SARS-CoV-2 detection. The solutions that rely on simultaneous use of CRISPR-Cas systems and isothermal amplification have also been commonly combined with a LFA readout (Zhu et al., 2021; Xiong et al., 2021). A dominant approach in the NALFA systems used for SARS-CoV-2 diagnosis is based on a colorimetric detection chemistry that comprises the interaction of oligonucleotide probes labeled with AuNPs and specific amplicons (Lau et al., 2021; Xiong et al., 2021). The amplicons are often generated in the sequence of the extension of primers labeled with biotin, which are then captured on commercially available strips by the anchoring system biotin-streptavidin (Azhar et al., 2021; Lau et al., 2021), producing an intense red signal. Alternative molecular reactions reported for detection of SARS-CoV-2 with NALFA are based on in-house developed variations of the above mentioned strategy (Zhang et al., 2021a; Zhu et al., 2021), or other type of solution involving different nanoparticles, as for instance carbon nanoparticles (CNPs) (Wu et al., 2021). CRISPR-Cas-based solutions request the incubation of the amplicons obtained by isothermal amplification with a Cas-crRNA that targets its sequence, activating the collateral cleavage activity of Cas. This sequence of events provokes the cleavage of reporter oligonucleotides, which are then read in the NALFA device, this way increasing the specificity of the detection step (Nguyen et al., 2020; Xiong et al., 2021).

### Microfluidic Devices

Microfluidics is based on the manipulation of fluids at a sub-millimeter scale, having multiple applications in molecular diagnostic with a growing number of solutions trusting on microfluidic-related platforms that contain nucleic acid amplification stages (Anderson et al., 2021; Fassy et al., 2021; Liu et al., 2021; Xing et al., 2021). The higher cost and complexity of these systems, which are often used at the point-of-care (**Table 7**), in comparison to LFA-based methods, are challenges that may discourage its use (Liu et al., 2021). Nonetheless, the processes of nucleic acid amplification and detection in microfluidics approaches may occur entirely in a closed system, rather than in a fragmented process always comprising closed-tube amplification and an open readout step, like it happens in methods that include LFA (de Oliveira et al., 2021; Liu et al., 2021). This fact points toward important advantages of microfluidic systems in avoiding contaminations, automatizing assays (Ramachandran et al., 2020), standardizing the different stages of reaction and enabling overall reduction in the time-to-result (de Oliveira et al., 2021; Liu et al., 2021). Microfluidic systems can be further divided in various subsets, of what rotationally-driven (Ji et al., 2020; Xiong et al., 2020; de Oliveira et al., 2021), electric field-driven (Ramachandran et al., 2020) and paper-based (Garneret et al., 2021) options were already adopted in the context of SARS-CoV-2. The normal workflow used for detecting viral RNA in other approaches, which includes extraction, amplification and detection of nucleic acids can be partially (de Oliveira et al., 2021; Fassy et al., 2021; Liu et al., 2021), or entirely (Garneret et al., 2021) conducted in microfluidic platforms, since the extraction step is often conducted out of the microfluics apparatus. There are different types of amplification strategies already assessed in these devices that rely on PCR-related methods, generally qRT-PCR (Anderson et al., 2021; Fassy et al., 2021; Xing et al., 2021), or isothermal amplification techniques related to LAMP (Ramachandran et al., 2020; Xiong et al., 2020; de Oliveira et al., 2021; Garneret et al., 2021), NASBA (Xing et al., 2020), RPA (Liu et al., 2021) and RCA (Kim et al., 2021), including approaches based on CRISPR-Cas (Ramachandran et al., 2020). In the context of SARS-CoV-2 diagnostics, the detection of DNA following the amplification reaction is usually performed by integrated measuring of fluorescence (Ramachandran et al., 2020; Xing et al., 2020; de Oliveira et al., 2021; Fassy et al., 2021; Garneret et al., 2021), although LFAs (Liu et al., 2021), or rheometry devices (Kim et al., 2021) have already been combined with microfluidic systems for this same purpose.

## Benchmarking of Commercial Diagnostics

### Criteria Used in the Assessment of Commercial Assays

The most frequent criteria used in the assessment and comparison of NAATs in review literature include distinct topics like the route of sample collection, the type of specimen, the processing strategy, the type of amplification method, or the detection mechanism, including both detection chemistry and detection platforms (Khan et al., 2020). Furthermore, other important parameters to be accessed are the duration of the entire process, detection range and the possibility to perform simultaneous detection of distinct amplicons (Khan et al., 2020). The certification of the diagnostic solution is an important aspect to take into account. There are well-established and as-fast-as-possible processes for verification of diagnostic assays prior to making them available in the market, during the current pandemic (Mitchell et al., 2020). These processes vary worldwide and, while European Union (EU) countries rely on CE mark for validating new *in-vitro* diagnostics (IVDs), CE-IVD (Vermeersch and André, 2021), United States trust on EUAs (Mitchell et al., 2020). EUAs are special authorizations that enable licensing the commercialization of a certain drug, IVD, or medical device under the legislation followed by FDA, despite the non-completion of the entire validation process (Mitchell et al., 2020). This type of authorization requests less data about product performance than regular authorizations, being valid only during the pandemic and ceasing after that state is deactivated (Mitchell et al., 2020). EUAs granted by Food and Drug Administration (FDA) are widely mentioned in research papers (Basu et al., 2020; Gorzalski et al., 2020; Smith et al., 2020), being trusted as an important seal of approval.

### Comparison of Different Groups of Commercial NAATs

PCR-related assays comprise standard RT-qPCR assays and other PCR-derived tests. Since it is impractical to analyze all assays granted with a EUA or other validation mark, by different regulatory bodies, a set of representative commercial assays issued with a EUA by FDA and/or CE-IVD mark by EU since December 2020 were chosen and benchmarked following the aforementioned criteria (**Table 8**). The analysis of the last mentioned table enables to verify that the most frequent route of collection among PCR-related assays relies on hetero-collection by healthcare workers, although there are options for self-collection, either with, or without supervision. URT specimens are the main source used for retrieving the RNA to be detected, being fit for all the tests assessed. NP samples are compatible with all the tests considered and BAL is the favorite LRT specimen to be collected. Processing of samples is almost always done through automated extraction, resorting to a full extraction approach. The range of amplification-based assays includes mainly RT-qPCR, although RT-ddPCR and qSTAR being also options and the targets being usually various sites, in multiplex protocols. The molecular mechanism of detection relies for the great part in real-time tracking of fluorescence, using specific oligonucleotide probes. While a significant number of tests are aimed for fully automated platforms, able to perform all testing process from sample processing to the interpretation of results, there are a greater number of tests designed for segmented analysis. Despite not being indicated in **Table 8**, the represented PCR-derived tests are all aimed to be performed at clinical settings.

**Table 8:** Benchmarking of diagnostics for SARS-CoV-2 based on non-isothermal amplification methods issued with EUA, or CE mark.

Tests	Producing company	Validation	Type of sample processing	Ready for Pooling	Amplification method	Detection strategy	Detection platform	Duration (min)	Detection limit (copies/µL)	Specimen under analysis	Targets, Multiplex, or Multi-species
**Cobas SARS-CoV-2**	Roche Molecular Systems, Inc	EUA, CE-IVD	Automated full extraction	Yes	RT-qPCR	Fluorescence, Taqman probes	Fully automated equipment	<210	0.046	Self-collected – URT swab (NS)//Collected by healthcare worker – URT swabs (NS,NP,OP)	Multi-target, ORF1ab and E and Multiplex
**NeuMoDx SARS-CoV-2 assay**	NeuMoDx Molecular, Inc	EUA, CE-IVD	Automated full extraction	No	RT-qPCR	Fluorescence, Taqman probes	Fully automated equipment	_	0.050 (saliva) or 0.150 (NP)	Supervised self-collection – saliva collected with NeuMoDx Saliva Collection Kit//Collected by healthcare worker - URT swabs (NS, NP, OP) and BAL	Multi-target, ORF1ab (NSP2) and N and Multiplex
**Xpert Omni SARS-CoV-2 Assay**	Cepheid	EUA, CE-IVD	Automated full extraction	No	RT-qPCR	Fluorescence, Nos-specified oligonucleotide hydrolysis probes	Fully automated equipment	_	0.4	Collected by healthcare worker – URT swabs (NS,NP,OP, mid-turbinate) and NS wash, or aspirate	Multi-target, N and E and Multiplex
**Bio-Rad Reliance SARS-CoV-2 RT-PCR Assay kit**	Bio-Rad Laboratories, Inc	EUA	Manual, or Automated full extraction	No	RT-qPCR	Fluorescence, No-specified oligonucleotide probes	qPCR equipment	_	0.125-0.250	Collected by healthcare worker – URT swabs (NS,NP, OP, mid-turbinate), nasal aspirates and nasal washes	Multi-target, two N gene regions and Multiplex
**BioFire COVID-19 Test**	BioFire Diagnostics,LLC	EUA	Automated full extraction	Yes	N-RT-qPCR	End-point Melting Curve data	qPCR equipment	50	5.4	Collected by healthcare worker – URT swabs (NS,OP, mid-turbinate), LRT samples (sputum, tracheal aspirates and BAL)	Multi-target, two ORF1ab regions and ORF8, Multiplex
**Lyra SARS-CoV-2 Assay**	Quidel, Inc	EUA, CE-IVD	Automated full extraction	No	RT-qPCR	Fluorescence, Taqman probes	qPCR equipment	135	6	Collected by healthcare worker – URT swabs (NP,OP)	Single-target, ORF1ab
**Quest SARS-CoV-2 rRT-PCR**	Quest Diagnostics Infectious Disease, Inc	EUA	Automated full extraction	Yes	RT-qPCR	Fluorescence, Taqman probes	qPCR equipment	_	0.136	Self-collected – URT swab (NS)//Collected by healthcare worker – URT (NP,OP) swabs and LRT samples (sputum, tracheal aspirates and BAL)	Multi-target, two N gene regions and Multiplex
**Quest SARS-CoV-2 rRT-PCR**	BioFire Diagnostics, LLC	EUA	Automated full extraction	No	N-RT-qPCR	End-point Melting Curve data	Fully automatedequipment	50	0.5 (SARS-CoV-2), in the range 0.01 to 3 for other pathogens	Collected by healthcare worker - URT swab (NP)	Multi-target, S and M (for SARS-CoV-2),Multiplex and Multi-species,
**Quest SARS-CoV-2 rRT-PCR**	Bio-Rad Laboratories, Inc	EUA	Manual or Automated full extraction	No	RT-ddPCR	Fluorescence, Taqman probes	Digital PCR equipment	<500	0.4	Collected by healthcare worker - URT swabs (NS, NP, OP, mid-turbinate), aspirates (NS,NP) and BAL	Multi-target, two N gene regions and Multiplex
**LumiraDx SARS-CoV-2 RNA STAR Complete**	Lumira Dx UK Ltd	EUA, CE-IVD	Brief lysis step	No	qSTAR	Fluorescence, Molecular beacons	qPCR equipment	<20	1.875	Collected by healthcare worker - URT swabs (NS, NP, OP, mid-turbinate) and BAL	Single-target, ORF1ab

All the information was retrieved from the instruction for use supplied in FDA website (https://www.fda.gov/medical-devices/coronavirus-disease-2019-covid-19-emergency-use-authorizations-medical-devices/in-vitro-diagnostics-euas-molecular-diagnostic-tests-sars-cov-2) and the data sheets attached to each test validated with CE-IVD, which are listed in European Comission website (https://covid-19-diagnostics.jrc.ec.europa.eu/devices?device_id=&manufacturer=&text_name=&marking=Yes&method=&rapid_diag=&target_type=&search_method=AND#form_content). Both sites were last accessed on 02.02.2022.

Regarding isothermal nucleic acid amplification tests, these are based in distinct reactions, being logic to analyze them in a separate group from PCR-related tests. Thus, a selection of tests granted with EUAs by FDA and/or CE-IVD mark by EU since December 2020 was made according with its distinct characteristics and benchmarked (**Table 9**). The most usual path for collection of samples and the main type of specimens for analysis is similar to those used in PCR-related assays. In general, processing of samples oscillates between full RNA extraction, either automated or manual, to partial extraction through a brief lysis step. The scope of isothermal amplification techniques included in this group of diagnostics is diverse, ranging from RT-LAMP and TMA, the two most popular nucleic acid amplification methods, to RT-HAD and other undisclosed procedures. There are plenty of molecular mechanisms of detection, including either end-point colorimetry, through color change analysis following pH shift upon amplification, real-time fluorescence tracking, with fluorescence-producing oligonucleotide probes, or real-time chemiluminescence measurement, through HPA. The platforms in which detection takes place are also diverse, including fully automated equipments, microfluidic platforms, LFA, plate readers, or single tube options. Overall, a great part of the tests displayed were designed as POCT, including Lucira CHECK-IT COVID-19 Test Kit, Detect COVID-19, Mobile Detect Bio BCC19 Kit, Solana SARS-CoV-2 Assay and ID NOW COVID-19, or can easily be compatible with a wide range of laboratory settings like Color SARS-CoV-2 RT-LAMP Diagnostic Assay, SHERLOCK CRISPR SARS-CoV-2 Kit and ALS SARS-CoV-2 RT-LAMP. Nevertheless, there are still alternatives for more complex and demanding instruments, like Procleix SARS-CoV-2 Assay and Aptima SARS-CoV-2 Assay. There are various isothermal amplification tests that have a shorter duration than PCR-based assays, often without compromising sensitivity, as evidenced by comparing **Tables 8** and **9**.

**Table 9:** Benchmarking of diagnostics for SARS-CoV-2 based on Isothermal amplification-derived methods issued with EUA, or CE mark.

Tests	Producing company	Validation	Type of sample processing	Amplification type	Detection mechanism	Detection platform	Duration (min)	Limit of detection (copies/µL)	Type of specimen under analysis	Targets, Mulltiplex, or Multi-species	Settings
**Lucira CHECK-IT COVID-19 Test Kit**	Lucira Health, Inc	EUA	Lysis step	RT-LAMP	Colorimetry, Color change induced by pH shift upon amplification, then converted in electronic signal	Microfluidics	30	0.9	Self-collected swab (NS)	Multi-target, two N gene regions and Multiplex	Home
**Detect ™ Covid-19**	Detect, Inc	EUA	Lysis step	RT-LAMP	Colorimetry, Visual observation of specific signal upon interaction of amplicons with colored NPs and retention of the conjugates on the test point	LFA	≈60	0.8	Self-collected swab (NS)	Single target, ORF1ab	Home
**MobileDetect Bio BCC19 Test Kit**	Mobile Detect Bio Inc	EUA	Non-specified processing reagents	RT-LAMP	Colorimetry, Visual detection of color change induced by pH shift upon amplification	Single-tube	<60	75	Collected by healthcare worker – URT swabs (NS,NP,OP, mid-turbinate)	Multi-target, N and E and Multiplex	Clinical
**Color SARS-CoV-2 RT-LAMP Diagnostic Assay**	Color Health, Inc	EUA	Automated full extraction	RT-LAMP	Colorimetry, Color change induced by pH shift upon amplification, then analysed spectrophotometrical	Micro-plate reader	70	0.75	Collected by healthcare worker – URT swabs (NS,NP,OP,mid-turbinate)	Multi-target, N and E, or ORF1ab,S and Multiplex	Clinical
**SHERLOCK ™ CRISPR SARS-CoV-2Kit**	Sherlock Biosciences, Inc	EUA	Manual full extraction	RT-LAMP	Fluorescence, Enzymatic-based cleavage of oligonucleotide probes containing a fluorophore and a quencher	Micro-plate reader	≈60	6	Collected by healthcare worker – URT swabs (NS,NP,OP), NS, NP aspirates and BAL	Multi-target, ORF1ab and N and Multiplex	Clinical
**ALS SARS-CoV-2 RT-LAMP**	ALS, Inc	CE -IVD	Non-specified, Non-conventional extraction	RT-LAMP	Colorimetry, Color change induced upon amplification	Single-tube	45	10	Collected by healthcare worker – URT samples	Multi-target, ORF1ab and N	Clinical
**Procleix SARS-CoV-2 Assay**	Grifols Diagnostic Solutions Inc	EUA	Automated full extraction	TMA	Chemiluminescence, using HPA	Fully automated equipment	_	0.06	Collected by healthcare worker – URT swabs (NS,NP,OP), NS, NP aspirates and BAL	Multi-target and Multiplex	Clinical
**Aptima SARS-CoV-2 Assay**	Hologic, Inc	EUA, CE-IVD	Lysis step	TMA	Chemiluminescence, using HPA	Fully automated equipment	_	0.6	Collected by healthcare worker – URT swabs (NS,NP, mid-turbinate)	Multi-target, two ORF1ab gene regions, Multiplex	Clinical
**Aptima SARS-CoV-2/Flu Assay**	Hologic, Inc	EUA	Lysis step	TMA	Fluorescence, similar to molecular beacons	Fully automated equipment	_	0.18	Collected by healthcare worker – URT swabs (NS,NP, OP, mid-turbinate), NS and NP aspirates	Multi-target, two ORF1ab gene regions, Multiplex and Multi-species	Clinical
**Solana SARS-CoV-2 Assay**	Quidel, Inc	EUA, CE-IVD	Lysis step and heat treatment	RT-HDA	Fluorescence, oligonucleotide probes hydrolysated by RNase H2	Fully automated equipment	30	54	Collected by healthcare worker – URT swabs (NS,NP)	Multi-target, ORF1ab	Clinical
**ID Now COVID-19**	Abbott Diagnostics Scarborough, Inc	EUA, CE-IVD	Lysis step	Unknown	Fluorescence, molecular beacons	Fully automated equipment	≈13	0.125	Collected by healthcare worker – URT swabs (NS,NP,TH)	Single-target, ORF1ab	Clinical

All the information was retrieved from the instruction for use supplied in FDA website (https://www.fda.gov/medical-devices/coronavirus-disease-2019-covid-19-emergency-use-authorizations-medical-devices/in-vitro-diagnostics-euas-molecular-diagnostic-tests-sars-cov-2) and the data sheets attached to each test validated with CE-IVD, which are listed in European Comission website (https://covid-19-diagnostics.jrc.ec.europa.eu/devices?device_id=&manufacturer=&text_name=&marking=Yes&method=&rapid_diag=&target_type=&search_method=AND#form_content). Both sites were last accessed on 02.02.2022.

## Conclusions and Near Future Perspectives

The diversity of testing strategies for the same application mirrors the widespread response of scientific community to the ongoing pandemic. The myriad of diagnostic approaches detailed during the body of this work denounces that the detection of SARS-CoV-2 in clinical samples resorting to NAATs can vary with the type of specimens, collection practices, method of extraction, process of amplification, detection chemistry and the type of platform where the analysis is conducted. While reaction variables are abundant, the types of controls used for each diagnostic approach are also diverse, what turns it difficult to compare distinct detection methods. The semi-quantitative nature of tests that is for the greatest part supported on Cq values (for the case of RT-qPCR-derived protocols) rather than in exact viral load numbers is also a factor that contributes for impairment in the comparison of test performances (Bustin et al., 2021; Cheema and Blumberg, 2021). Therefore the standardization of NAATs, as well as reaction controls, aimed at SARS-CoV-2 diagnosis is an issue that continues to demand attention (Bustin et al., 2021; Cheema and Blumberg, 2021).

POCTs rely on low budget solutions to proportionate wide access to essential diagnosis needs, turning these type of tests very useful in the pandemic scenery (Silva et al., 2021). However, despite countless works devoted to demonstrate the ability of NAAT-based approaches to be used as POCTs, the wide number of solutions wasn´t enough to dislodge RT-qPCRs as the prime choice for performing SARS-CoV-2 detection (Bustin et al., 2021). In addition to the robustness and widespread use that is associated with RT-qPCR, the fact that most of POCTs rely on isothermal nucleic acid amplification, which are not mature technologies in the market, may explain the back position of this type of diagnostics (Mattioli et al., 2020). However, isothermal POCTs are gaining more and more traction. The continuous development of nanotechnology-based solutions that can turn isothermal amplification strategies more robust in terms of sensitivity and can promote fast adaptation to detection of rising variants will benefit these type of tests. Miniaturized PCR-based systems also haven´t substituted traditional laboratory-based diagnostics, although good prospects exist of growing utilization (Gupta et al., 2021). Furthermore, the articulation of simple-functioning detection platforms with digital tools, originating faster measurements, interpretation and delivery of test results are good assets in any POCT.

Despite the efficient testing campaigns largely based on RT-qPCR, the potential role of POCTs as an alternative to PCR and the emergence of more robust POCT platforms would enable to better cope with a future pandemic. Also, new surging variants may challenge health systems in less favored regions, thus stressing the need for efficient POCTs. This way, more efforts need to be put in advancing POCTs for SARS-CoV-2 detection.

## Author Contributions

JV drafted the article, assembled and performed the analysis of data. EP, NA, and CA critically revised the article, enhancing the overall work. All authors contributed to the article and approved the submitted version.

## Funding

This work was financially supported by LA/P/0045/2020 (ALiCE), UIDB/00511/2020 and UIDP/00511/2020 (LEPABE), funded by national funds through FCT/MCTES (PIDDAC); Project FCT_128_596771122 (RESEARCH 4 COVID-19), funded by national funds (PIDDAC) through FCT/MCTES. The authors also thank FCT for the PhD Fellowship 2020.10243.BD.

## Conflict of Interest

The authors declare that the research was conducted in the absence of any commercial or financial relationships that could be construed as a potential conflict of interest.

## Publisher’s Note

All claims expressed in this article are solely those of the authors and do not necessarily represent those of their affiliated organizations, or those of the publisher, the editors and the reviewers. Any product that may be evaluated in this article, or claim that may be made by its manufacturer, is not guaranteed or endorsed by the publisher.
